# Identification of the *PFK* gene family in *Solanum* species and expression analysis in the fruitof *Solanum lycopersicum*

**DOI:** 10.3389/fgene.2026.1738448

**Published:** 2026-03-09

**Authors:** Zepeng Wang, Zhongyu Wang, Ruiqiang Xu, Qingyuan Meng, Jintao Wang, Ning Li, Qinghui Yu

**Affiliations:** 1 College of Horticulture, Xinjiang Agricultural University, Urumqi, China; 2 Biological Breeding Laboratory, Xinjiang Uygur Autonomous Region Academy of Agricultural Sciences, Urumqi, China; 3 Institute of Vegetables and Flowers, Chinese Academy of Agricultural Sciences, Beijing, China

**Keywords:** expression analysis, fruit development, PFK gene family, sugar metabolism, tomato pangenome

## Abstract

**Introduction:**

Phosphofructokinase (PFK) is a crucial rate-limiting enzyme in glycolysis, essential for sugar metabolism and fruit quality. This study provides the first pangenome-scale analysis of the *PFK* family across *Solanum* species.

**Methods:**

Using pan-genome data, 156 *PFK* genes were identified across 12 *Solanum* species. Comprehensive bioinformatic analyses, protein-protein interaction predictions, and promoter motif scans were performed. Expression patterns across four fruit developmental stages were characterized via RNA‐seq and validated by qRT‐PCR.

**Results:**

The *PFK* family, categorized into PFK and PFP subfamilies, expanded primarily through segmental duplication under strong purifying selection. We identified distinct, stage-specific expression patterns, with *SolyPFK07* and *SolyPFPA2* emerging as key regulators of sugar accumulation. Promoters contained numerous elements responsive to hormones and abiotic stresses.

**Conclusion:**

*PFK* genes are vital for fruit development, sugar metabolism, and stress adaptation. These findings offer a theoretical basis and genetic resources for the molecular breeding of high-quality tomatoes.

## 1 Introduction

Sugars are not only the primary energy source for plant growth but also serve as crucial signaling molecules that regulate growth and development in higher plants (Lastdrager et al., 2014; Lü et al., 2019). Phosphofructokinase (PFK), a key rate-limiting enzyme in glycolysis, plays an essential role in plant sugar metabolism. In plants, two types of phosphorylating enzymes are typically involved in the phosphorylation process. One is ATP-dependent phosphofructokinase (PFK, EC 2.7.1.11), which catalyzes the irreversible phosphorylation of fructose-6-phosphate (F-6-P) to fructose-1,6-bisphosphate (F-1,6-BP). The other is pyrophosphate-dependent fructose-6-phosphate phosphotransferase (PFP, EC 2.7.1.90), which catalyzes the reversible conversion of F-6-P to F-1,6-BP (Bapteste et al., 2003; Lim et al., 2014; Siebers and Schönheit, 2005). Generally, PFP is composed of α- and β-subunits, where the α-subunit regulates catalytic activity via fructose-2,6-bisphosphate (Fru-2,6-BP), while the β-subunit performs the catalytic function (Chen et al., 2020a; Qin et al., 2014).

With the completion of whole-genome sequencing in multiple plant species, the *PFK* gene family has been increasingly investigated. In Arabidopsis (*Arabidopsis thaliana*), 11 *PFK* genes have been identified, including seven members belonging to the PFK subfamily and four members belonging to the PFP subfamily (Mustroph et al., 2007). In cassava (*Manihot esculenta*), 13 *PFK* genes have been identified, comprising nine *MePFK* subtype genes and four *MePFP* subtype genes, among which *MePFPA1* plays a prominent role under waterlogging stress (Wang et al., 2021). In four cotton species (*Gossypium hirsutum*, *Gossypium barbadense*, *Gossypium arboreum*, and *Gossypium raimondii*), a total of 80 *PFK* genes have been identified, including 56 genes from the PFK subfamily and 24 genes from the PFP subfamily (six PFPA and 18 PFPB genes). Notably, *GhPFK11* and *GhPFK17* have been identified as major candidate genes involved in drought tolerance regulation in cotton (Mehari et al., 2022). In wheat (*Triticum aestivum*), 24 *TaPFK* subtype genes and 12 *TaPFP* subtype genes have been identified, with the *TaPFP* genes playing a dominant role in growth and development (Yu et al., 2021). In rice (*Oryza sativa*), 15 distinct sequences with high similarity to PFK-encoding genes in Arabidopsis have been identified (Mustroph et al., 2013). In maize (*Zea mays*), a total of 18 *ZmPFK* genes have been identified, and functional analysis revealed that members of the *ZmPFK* family are crucial in response to low-temperature stress, with *ZmPFK2*, *ZmPFK6*, and *ZmPFK18* playing essential roles in maize growth, development, and stress responses (Fu et al., 2025). In addition, comprehensive genome-wide identification of *PFK* genes has also been conducted in several other crops, including pear (*Pyrus bretschneideri*), spinach (*Spinacia oleracea*), sugarcane (*Saccharum officinarum*), red oak (*Quercus rubra*), and eastern cottonwood (*Populus deltoides*) (Lü et al., 2019; Chen et al., 2017; Zhu et al., 2013; Kim et al., 2023a; Kim et al., 2023b). Collectively, these studies indicate that the *PFK* gene family has been widely investigated, and its regulatory functions are gradually being uncovered and experimentally validated.

Tomato (*Solanum lycopersicum*) is one of the most important vegetable crops worldwide. It is extensively cultivated in many regions due to its high economic and nutritional value and is highly favored by both markets and consumers (Wang et al., 2023). At present, research on fruit quality traits has become a primary focus in tomato breeding programs, particularly for processing tomatoes. Sugar content plays a crucial role in determining tomato fruit quality. During tomato development, from flowering to full ripening, sugar accumulation is regulated by both endogenous and exogenous signals, primarily mediated through hormone and sugar signaling pathways, ultimately affecting fruit flavor and quality at the red-ripe stage (Baldet et al., 2006; Mounet et al., 2009). Previous studies have shown that *MdPFPβ* enhances photosynthetic performance in transgenic tomato plants, modulates the activities of sugar metabolism-related enzymes, and promotes the accumulation of sucrose, fructose, and glucose in fruits, suggesting that *PFK* genes are involved in the regulation of sugar metabolism in tomato (Yu et al., 2022). Currently, cultivated tomato has lost a considerable portion of its genetic diversity due to domestication bottlenecks and intensive artificial selection for larger fruits and higher yields. In contrast, wild tomato species exhibit broad genetic and phenotypic diversity. For example, *S. pennellii* not only contains higher sugar levels but also displays stronger resistance to biotic and abiotic stresses compared with cultivated tomato; *S. peruvianum* provides valuable resistance resources against various diseases, including Septoria leaf spot, Fusarium wilt, leaf mold, and late blight; *S. pimpinellifolium* possesses a rich flavor that is three times stronger than cultivated tomato and is also enriched in vitamin C; and *S. neorickii* has a high soluble solids content (Dai et al., 2022; Zhu, 2015). Therefore, the continued exploitation of wild tomato germplasm remains of great practical significance for breeding high-quality tomato varieties.

The assembly and release of the tomato pan-genome have substantially enhanced the ability of breeders to integrate and exploit the abundant genetic diversity found in wild tomato species (Li et al., 2023). In this context, we conducted a genome-wide identification and expression analysis of the *PFK* gene family across 12 tomato species, resulting in the identification of 156 *PFK* genes. Comprehensive analyses, including phylogenetic tree construction, gene structure characterization, motif identification, Chromosomal distribution, promoter element prediction, and protein–protein interaction network analysis, were performed. Furthermore, RNA-seq and qRT-PCR approaches were combined to examine the expression patterns of *PFK* genes in relation to sugar content during four stages of fruit development, thereby elucidating their potential roles in sugar accumulation dynamics. The findings of this study establishes a theoretical basis for the in-depth analysis of the function of the *PFK* gene in the growth and development of tomato fruit and its sugar metabolism. This is highly valuable for creating high-quality tomato germplasm resources and breeding high-quality tomatoes.

## 2 Materials and methods

### 2.1 Plant materials

The Tomato Genetics Resource Center (TGRC) supplied the processing tomato cultivar 'E−6203′that was used in this study. Seeds were sown in seedling trays and grown in a greenhouse at 25 °C for cultivation. When the seedlings reached the four-leaf stage, they were transplanted to the experimental field of the Bazhou Academy of Agricultural Sciences, Korla City, Xinjiang, China. The early developmental stage of tomato fruit is characterized by active cell division and cell enlargement. The ripening and maturation stages of tomato fruit include the mature green stage, the breaker stage, and the red ripe stage, during which the fruit size and shape remain largely unchanged, while the fruit color and metabolic composition undergo dramatic changes. Tomato fruits were harvested at 15 days after pollination (fruit expansion stage), 40 days (mature green stage), 49 days (breaker stage), and 59 days (red ripe stage). For each plant, 3–5 fruits were collected and immediately frozen in liquid nitrogen. A portion of the samples was used for RNA extraction, while the remaining samples were stored at −80 °C for future use.

### 2.2 Identification of PFK gene family members

To identify potential members of the *PFK* gene family in *Solanum* species, genome-wide data from the tomato pan-genome generated in our previous study were used to screen for *PFK* genes in each species independently (Li et al., 2023). Additionally, genome sequences of kiwifruit (*Actinidia chinensis*), apple (*Malus domestica*), watermelon (*Citrullus lanatus*), peach (*Prunus persica*), potato (*Solanum tuberosum*), grape (*Vitis vinifera*), maize (*Zea mays*) and arabidopsis (*Arabidopsis thaliana*) were downloaded from the Ensembl database (https://plants.ensembl.org/). The hidden Markov model (HMM) profile of the PFK domain (Pfam ID: PF00365) was retrieved from the InterPro database (http://pfam-legacy.xfam.org/) and used as a query in HMMER searches against the predicted protein sequences of tomato PFK genes, with an E-value cutoff of ≤1e^−5^ (Prakash et al., 2017). Finally, all candidate proteins were further validated using the Conserved Domain Database (CDD) tool at NCBI (https://www.ncbi.nlm.nih.gov/).

### 2.3 Physicochemical properties, secondary structure prediction, and subcellular localization of PFK proteins

The physicochemical properties of tomato PFK proteins were analyzed using the ExPASy online tool (https://web.expasy.org/tools/) (Gasteiger et al., 2003). The secondary structures of all PFK proteins were predicted with the SOPMA program (https://npsa-pbil.ibcp.fr/cgi-bin/npsa_automat.pl?page=npsa_sopma.html) and subcellular localization was assessed using WoLF PSORT II (https://wolfpsort.hgc.jp/), in order to predict their potential functional compartments (Geourjon and Deléage, 1995).

### 2.4 Phylogenetic analysis of PFK genes

PFK protein sequences from tomato, kiwifruit, apple, potato, grape, maize, and arabidopsis were aligned using MUSCLE implemented in MEGA 7.0. A phylogenetic tree of the *PFK* gene family was constructed using the Neighbor-Joining (NJ) method with 1,000 bootstrap replicates to assess branch reliability (Kumar et al., 2018). The resulting tree was subsequently visualized and refined using the EVOLVIEW online platform (https://www.evolgenius.info/evolview-v2/) (Zhang et al., 2012).

### 2.5 Gene structure analysis of PFK genes

To analyze the structural characteristics of *PFK* genes, the coding sequences (CDS) were compared with their corresponding genomic DNA sequences using TBtools. Conserved motif prediction was performed with the MEME online suite (https://meme-suite.org/meme/), with the maximum number of motifs set to 20, motif widths ranging from 6 to 100 amino acids, and an E-value threshold of 1e-5. (Ba et al., 2009). Finally, the phylogenetic relationships, gene structures, conserved protein motifs, and protein domains of the PFK gene family were integrated and visualized using TBtools (Chen et al., 2020b).

### 2.6 Chromosomal localization, collinearity, and Ka/Ks analysis of PFK genes

Based on the Chromosomal location information of *PFK* genes, their positional distribution was visualized using TBtools. MCScanX was employed to generate collinearity maps of PFK genes among tomato, kiwifruit, watermelon, apple, peach, potato, and grape, as well as to analyze collinearity within *Solanum* species (Wang et al., 2012). Circos was used to visualize Chromosome-level collinearity relationships (Krzywinski et al., 2009). The Ka/Ks ratios of collinear *PFK* gene pairs in tomato were calculated using the Ka/Ks tool (Wang et al., 2010).

### 2.7 Promoter cis-acting element analysis of PFK genes

The 2,000 bp upstream sequences of *PFK* genes were extracted using TBtools and submitted to the PlantCARE online database (http://bioinformatics.psb.ugent.be/webtools/plantcare/html/) to predict cis-acting regulatory elements in the promoter regions (Lescot et al., 2002). Expression heatmaps of PFK genes were generated using the HeatMap tool in the ChiPlot online platform (https://www.chiplot.online/).

### 2.8 Expression pattern analysis of PFK genes

Transcriptome data were used to investigate the expression patterns of *PFK* genes during different developmental stages of tomato fruit, these data were downloaded from the Tomato Functional Genomics Database (TFGD; http://132.236.156.160/cgi-bin/TFGD/digital/home.cg). Expression heatmaps of *PFK* genes were generated using the HeatMap module in TBtools, and the resulting figures were refined with Adobe Illustrator 2020 for visualization.

### 2.9 Protein–protein interaction analysis of PFK proteins

Protein–protein interaction networks of PFK proteins were predicted using the STRING online database (https://string-db.org/), and the interaction networks were subsequently visualized with Cytoscape software (Szklarczyk et al., 2018; Su et al., 2014).

### 2.10 qRT-PCR analysis of PFK genes

Total RNA was extracted using the RNAprep Pure Plant Kit (DP441, Tiangen Biotech, Beijing, China). First-strand cDNA was synthesized with the All-In-One 5× RT MasterMix kit (abm, Canada). Each qRT-PCR reaction was performed in a 20 μL volume containing 10 μL of 2× ChamQ Universal SYBR qPCR Master Mix (Vazyme, China), 8.2 μL of ddH_2_O, 0.4 μL of each gene-specific primer, and 1 μL of cDNA template. Amplifications were performed on a LightCycler 96 Real-Time PCR System (Roche, Switzerland) with the following program: 94 °C for 120 s (pre-denaturation), followed by 45 cycles of 94 °C for 5 s (denaturation), 15 s for annealing, and 72 °C for 10 s (extension). Gene-specific primers for members of the tomato *SolyPFK* gene family were designed based on their CDS sequences (Table 1) and synthesized by Sangon Biotech (Shanghai, China). Relative expression levels were calculated using the 2^−ΔΔCT^ method, with *SolyActin* serving as the internal control. All experiments were conducted with three biological replicates.

**TABLE 1:** Primer sequences for qRT-PCR experiments.

Gene	Forward primer sequence (5'→ 3′)	Reverse primer sequence (5'→ 3′)
*SolyPFPB1*	GCA​GTG​GTA​GGG​ACA​AGA​TTG​A	GGC​ATC​CAA​TAA​CCC​GAG​TT
*SolyPFK01*	TGG​CTT​TGA​TAC​TGC​TGT​GGA​G	CAA​CAG​TCC​ACA​TCA​CGG​TTA​C
*SolyPFK02*	ATG​CTC​TTT​GTG​CTG​GGT​GG	GCT​TCT​TCA​ACG​GCA​GTA​TCA​A
*SolyPFK03*	TGT​GCC​CTG​GGA​TGA​ATA​CG	CCA​ATC​ATC​AAC​CAT​CTT​CGG
*SolyPFK04*	TAA​TGG​GAC​GGG​ATA​GTG​GG	TGT​GTC​CAT​TCT​CTT​TCA​GCC
*SolyPFPA1*	GTG​GTC​CTG​GTA​AAG​CAT​CAA​T	GGG​TAA​CAG​CCT​TGG​CAT​CT
*SolyPFK05*	CGG​TTG​GTC​CTG​TCA​ATA​ACA	GAG​TAG​GTG​GTT​CTT​CGT​CCT​TG
*SolyPFPB2*	CAA​CCG​TCT​CAT​TGT​ATC​GCT	CCT​GGA​AGT​AAC​GAC​ACG​GA
*SolyPFK06*	CAG​AGT​CAC​CCT​TCT​TTC​TTG​ATG	CTT​GTT​GAC​TTC​CAG​CAT​TGG
*SolyPFK07*	TGT​TGA​AGA​AGC​ACA​GAG​AGC​C	AAA​GGC​ACC​TCT​GGA​ATC​AAG
*SolyPFPA2*	CAT​CCA​GCC​ACT​GTA​GAT​TTG​A	TCC​TGG​TCT​TCA​ACG​CAA​A
*SolyPFK08*	GGA​GCA​ATG​GCA​GGA​TAC​AC	CTT​GGC​TGA​TTG​GTT​GAC​G
*SolyActin*	CAG​GGT​GTT​CTT​CAG​GAG​CAA	GGT​GTT​ATG​GTC​GGA​ATG​GG

## 3 Results

### 3.1 Physicochemical characterization of PFK genes

In this study, a total of 156 putative *PFK* genes were identified across 12 *Solanum* species (cultivated and wild tomatoes) via genome-wide identification analysis., including *S. lycopersicum* (12), *S. habrochaites* (12), *S. chilense* (14), *S. chmielewskii* (13), *S. corneliomulleri* (13), *S. galapagense* (13), *S. lycopersicoides* (16), *S. lycopersicum* var. *cerasiforme* (13), *S. neorickii* (13), *S. pennellii* (12), *S. peruvianum* (12), and *S. pimpinellifolium* (13). Genes were named according to their chromosomal locations and subfamily classifications. Analysis of physicochemical properties (Table 2; Supplementary Tables S1-S11) revealed that the amino acid lengths of *PFK* family members ranged from 273 residues (*SpenPFK06*) to 644 residues (*SlydPFPA1*), with molecular weights ranging from 30,139.41 Da (*SpenPFK06*) to 70,739.20 Da (*SlydPFPA1*). The predicted isoelectric points (pI) varied between 5.05 (*SlydPFK05*) and 8.95 (*SlydPFK02*). The GRAVY (grand average of hydropathicity) values ranged from −0.339 (*SlyvPFK09*) to −0.086 (*SchiPFPA1*), indicating that all *PFK* family members are hydrophilic proteins.

**TABLE 2:** Physicochemical properties of amino acid sequences encoded by members of the *PFK* gene family in *S. lycopersicum*.

Gene ID	Gene name	Chromosome location	Amino acid	Molecular weight	Isoelectrc point	Gravy	Subcellular localization	Protein secondary structure		
								a	b	c
Solyc02g081160.3.1	*SolyPFPB1*	Soly-Chr02	569	61765.81	6.01	−0.134	Cyto	44.46%	5.98%	33.92%
Solyc03g093520.3.1	*SolyPFK01*	Soly-Chr03	539	59714.13	8.71	−0.265	Chlo	31.91%	6.68%	42.49%
Solyc04g014270.3.1	*SolyPFK02*	Soly-Chr04	533	58460.03	8.55	−0.133	Cyto	31.89%	8.26%	41.84%
Solyc04g015200.3.1	*SolyPFK03*	Soly-Chr04	456	50513.51	6.31	−0.161	Chlo	31.80%	8.11%	40.13%
Solyc04g072580.1.1	*SolyPFK04*	Soly-Chr04	499	54851.02	5.20	−0.234	Cyto	31.26%	7.41%	43.09%
Solyc04g082880.4.1	*SolyPFPA1*	Soly-Chr04	616	67301.18	6.76	−0.112	Cyto	46.59%	5.52%	33.93%
Solyc07g045160.3.1	*SolyPFK05*	Soly-Chr07	509	56188.95	6.25	−0.230	Cysk	33.99%	7.47%	42.04%
Solyc07g049280.3.1	*SolyPFPB2*	Soly-Chr07	554	60090.89	7.56	−0.169	Chlo	44.04%	5.78%	34.12%
Solyc08g066100.3.1	*SolyPFK06*	Soly-Chr08	500	55053.79	6.92	−0.197	Cyto	33.80%	7.80%	41.60%
Solyc11g010450.2.1	*SolyPFK07*	Soly-Chr11	532	58365.79	6.47	−0.104	Cyto	30.45%	7.33%	43.80%
Solyc12g095760.2.1	*SolyPFPA2*	Soly-Chr12	617	67267.19	7.23	−0.153	Cyto	46.35%	5.51%	34.85%
Solyc12g095880.2.1	*SolyPFK08*	Soly-Chr12	485	53800.03	6.12	−0.339	Cyto	31.96%	8.04%	40.21%

Cyto, Cysk, and Chlo refer to cytoplasm, cytoskeleton, and chloroplast, respectively. The same abbreviations apply throughout.

Subcellular localization analysis revealed that members of the *PFK* gene family were predominantly distributed in the cytoplasm, chloroplast, and cytoskeleton. Specifically, *PFK* genes on Chr08 and Chr12 were localized to the cytoplasm and cytoskeleton, whereas all *PFK* genes on Chr11 were exclusively localized to the cytoplasm. The *PFK* genes on Chr03 were found in the chloroplasts, while those on Chr02 were distributed in both the cytoplasm and chloroplasts. *PFK* genes on the remaining chromosomes were observed in the cytoplasm, chloroplasts, and cytoskeleton.

The predicted secondary structure of proteins encoded by the *PFK* gene family was primarily composed of α-helices, β-sheets, and random coils. Among these, α-helices (28.25% in *SlydPFK11*% to 47.90% in *SchiPFPA1*) and random coils (31.93% in *SlydPFPB1* to 46.43% in *SneoPFK08*) represented the major structural components, whereas β-sheets were comparatively less abundant, ranging from 4.21% (*SlydPFPA2*) to 10.26% (*SpenPFK06*).

### 3.2 Chromosomal localization of PFK genes

**FIGURE 1:**
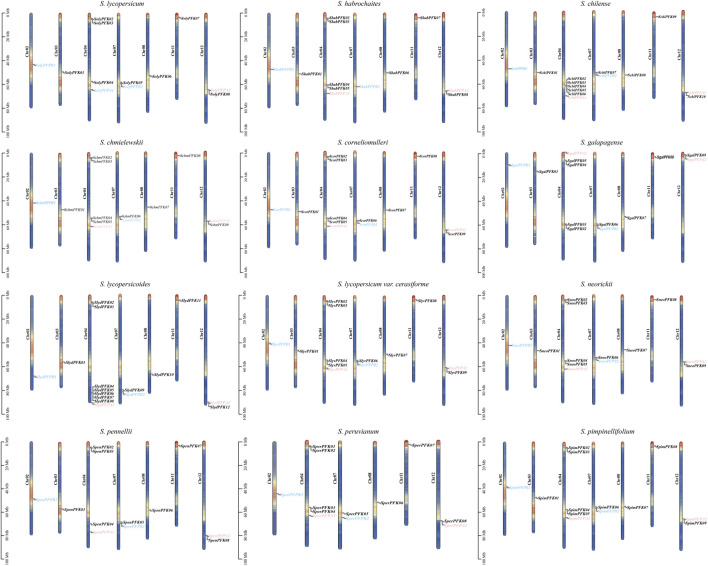
Chromosomal localization of the *PFK* gene. The names of the chromosomes are shown on the left of each Chromosome. The black line indicates the gene location. The five colors on the Chromosome, dark blue, light blue, yellow, light red, and dark red, represent the gene density, with red representing high levels and blue representing low levels. The size of the Chromosome is listed in metabases (Mb).

Chromosomal localization analysis revealed that all tomato *PFK* gene family members were distributed across seven chromosomes (Figure 1). Among them, the largest number of *PFK* genes was located on Chr04 (62), followed by Chr12 (24) and Chr07 (23). The remaining genes were distributed on Chr02 (12), Chr03 (11), Chr08 (12), and Chr11 (12). In the tomato *PFK* gene family, members of the PFK subfamily were mapped to Chr03, Chr04, Chr07, Chr08, Chr11, and Chr12; the PFP-α subfamily was distributed on Chr04 and Chr12; and the PFP-β subfamily was distributed on Chr02 and Chr07. Notably, only *S. peruvianum* lacked *PFK* genes on Chr03, whereas all other Solanum species had PFK genes distributed on Chr02, Chr03, Chr04, Chr07, Chr08, Chr11, and Chr12.

### 3.3 Phylogenetic classification of PFK genes

**FIGURE 2:**
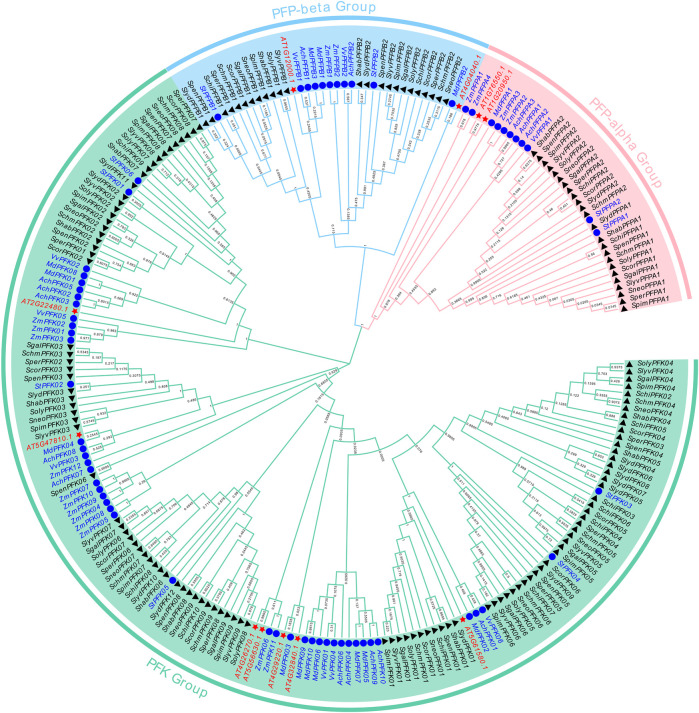
The phylogenetic tree and classification of *PFK* gene. In the phylogenetic tree, species are distinguished by different colored shapes: black triangles indicate tomato and its wild relatives (*S. lycopersicum*, *S. habrochaites*, *S. chilense*, S. chmielewskii, S. corneliomulleri, S. galapagense, *S. lycopersicoides*, *S. lycopersicum var. cerasiforme*, *S. neorickii*, S. pennellii, S. peruvianum, and *S. pimpinellifolium*); blue circles represent *Actinidia chinensis*, *Malus domestica*, *Solanum tuberosum*), *Vitis vinifera*), and *Zea mays*; and red stars represent *Arabidopsis thaliana*.

To elucidate the evolutionary relationships of *PFK* genes across different species, a phylogenetic tree was constructed based on the conserved protein domains of PFK from kiwifruit (*AchPFK*, 15), apple (*MdPFK*, 14), potato (*StPFK*, 10), grape (*VvPFK*, 9), maize (*ZmPFK*, 18), arabidopsis (*AtPFK*, 11), and tomato (*SlPFK*, 156) (Figure 2). In *Arabidopsis thaliana*, At1g20950 and At1g76550 were identified as the PFP-α subunits, while At1g12000 and At4g04040 were designated as the PFP-β subunits. Utilizing the Arabidopsis protein sequences along with those from six other plant species, a phylogenetic tree was constructed. Based on the distribution of Arabidopsis subunits, the *PFK* genes from these seven species were divided into two major subfamilies: the PFK subfamily and the PFP subfamily. The PFP subfamily was further resolved into PFP-α and PFP-β clades. Members within the same subfamily exhibited closer phylogenetic relationships. The distribution of members across subfamilies was uneven. The PFK subfamily contained the largest number of members (159), including 10 *AchPFKs*, 10 *MdPFKs*, 6 *StPFKs*, 6 *VvPFKs*, 12 *ZmPFKs*, 7 *AtPFKs*,and 108 *SlPFKs*. The PFP-α subfamily comprised 37 members, including 3 *AchPFKs*, 1 *MdPFK*, 2 *StPFPs*, 1 *VvPFP*, 4 *ZmPFKs*, 2 *AtPFKs*, and 24 *SlPFKs*. Similarly, the PFP-β subfamily also consisted of 37 members, including 2 *AchPFKs*, 3 *MdPFKs*, 2 *StPFPs*, 2 *VvPFPs*, 2 *ZmPFKs*, 2 *AtPFKs*, and 24 *SlPFKs*. Overall, genes within the same subfamily generally shared conserved functional characteristics, suggesting that members may have retained similar biochemical roles during evolution. Notably, most tomato *PFK* genes were clustered within the PFK subfamily, whereas a smaller proportion were assigned to the PFP subfamily, implying that sugar metabolism in tomato fruits is primarily regulated through the PFK-mediated pathway.

### 3.4 Motif composition and gene structure of PFK genes

**FIGURE 3:**
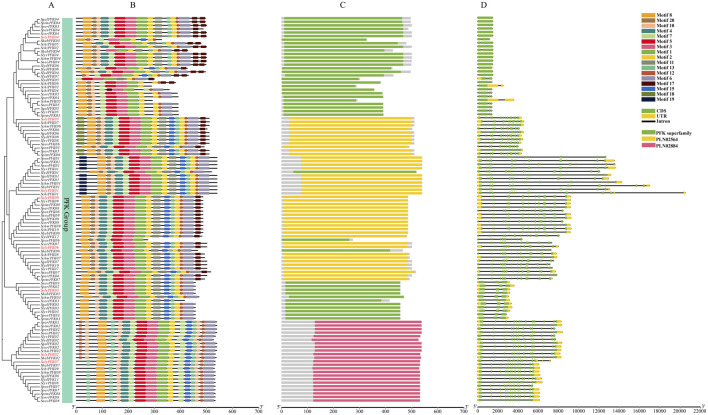
Features of the *PFK* genes in PFK subfamily. **(A)** Phylogenetic tree of PFK proteins in *Solanum* species. **(B)** Distribution of conserved motifs across *Solanum* species PFK proteins. **(C)** The domains of *Solanum* species PFK proteins. **(D)** Schematic representation of the gene structure of *Solanum* species *PFK* genes.

**FIGURE 4:**
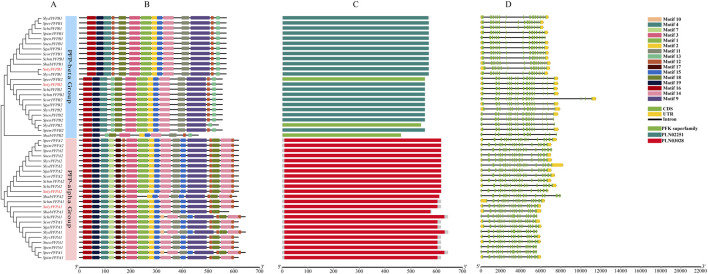
Features of the *PFK* genes in PFP subfamily. **(A)** Phylogenetic tree of PFK proteins in *Solanum* species. **(B)** Distribution of conserved motifs across *Solanum* species PFK proteins. **(C)** The domains of *Solanum* species PFK proteins. **(D)** Schematic representation of the gene structure of *Solanum* species *PFK* genes.

Exon–intron structural variation represents an important source of gene family diversification and plant biodiversity. As shown in Figure 3, within the PFK subfamily, *SgalPFK04*, *SpimPFK04*, *SperPFK03*, *SpenPFK04*, *ScorPFK04*, *SolyPFK04*, *SchiPFK02*, *SlyvPFK04*, *SchmPFK04*, *SneoPFK04*, *SlydPFK06*, and *SlydPFK04* contained only a single exon. *SchiPFK05* and *ShabPFK04* harbored two exons, whereas *SlydPFK05*, *SneoPFK03*, *SperPFK02*, *SolyPFK03*, *ShabPFK03*, *SchmPFK03*, *ScorPFK03*, *SgalPFK03*, *SlydPFK03*, *SlyvPFK03*, *SpenPFK03*, and *SpimPFK03* each contained three exons, indicating considerable variation in gene structure. As shown in Figure 4, members of the PFP subfamily generally contained a larger number of exons. Moreover, gene structures within the same subfamily exhibited both conserved and divergent regions. Even when exon numbers were identical, gene length often varied among homologs. For example, *SolyPFK03* and *SperPFK02* in the PFK subfamily both contained three exons, but differed in gene length. Similarly, *SolyPFPA2* and *SchmPFPA2* within the PFP-α subfamily both contained twenty exons, yet showed variations in both gene length.

PFK proteins exhibited diverse motif types, with functionally similar proteins typically sharing conserved Motif of similar composition and arrangement. Within the PFK subfamily, most members contained highly similar motif compositions and arrangements, including Motif 8, Motif 20, Motif 10, Motif 4, Motif 7, Motif 5, Motif 3, Motif 1, Motif 2, Motif 11, Motif 15, Motif 13, Motif 12, Motif 6, and Motif 17. In contrast, Motif 18 and Motif 19 were only present in a subset of members, suggesting that these genes may perform specialized functions. In the PFP-α subfamily, most members exhibited a conserved motif pattern consisting of Motif 16, Motif 19, Motif 4, Motif 13, Motif 18, Motif 3, Motif 1, Motif 2, Motif 15, Motif 14, Motif 11, Motif 9, and Motif 12. Similarly, members of the PFP-β subfamily shared a conserved arrangement of Motif 16, Motif 19, Motif 4, Motif 7, Motif 17, Motif 12, Motif 3, Motif 1, Motif 2, Motif 15, Motif 14, Motif 11, Motif 9, and Motif 18. Although the PFP-α and PFP-β subfamilies shared the majority of Motif, certain Motif were specific to one subgroup. For example, Motif 13 was unique to the PFP-α subfamily, whereas Motif 17 was only present in the PFP-β subfamily, indicating potential involvement in distinct regulatory functions.

Notably, all members of both the PFK and PFP subfamilies contained Motif 1 and Motif 3, suggesting that these Motif are widely distributed and highly conserved across tomato PFK proteins. Many members within the same subfamily also shared additional conserved Motif, supporting the functional conservation of these proteins. Meanwhile, some Motif were specific to certain subfamilies: Motif 6, Motif 8, and Motif 20 were restricted to the PFK subfamily, whereas Motif 9, Motif 14, and Motif 16 were unique to the PFP subfamily. These results indicate that PFK proteins exhibit a combination of conserved and subfamily-specific motifs, reflecting both structural conservation and functional diversity. Protein domain analysis revealed five distinct domain types across all tomato PFK proteins: the PFK superfamily, PLN02251, PLN02564, PLN02884, and PLN03028. Within the PFK subfamily, some members contain a single PFK superfamily domain, while others possess only the PLN02564 domain. Notably, a small subclade within this subfamily is characterized exclusively by the presence of the PLN02884 domain. The PFP subfamily primarily involves three domain types: the PFK superfamily, PLN02251, and PLN03028. Specifically, all members of the PFP-α subfamily exclusively contain the PLN03028 domain. In the PFP-β subfamily, proteins contain either the PFK superfamily or PLN02251 domain; only three members (SperPFPB2, SlydPFPB2, and ShabPFPB2) harbor a single PFK superfamily domain, whereas the remaining members exclusively contain the PLN02251 domain. These findings suggest that the diversity in domain composition among tomato PFK proteins reflects functional specialization within the family, providing a structural basis for their distinct roles in glycolysis and related metabolic pathways.

### 3.5 Synteny and Ka/Ks analysis of PFK genes

**FIGURE 5:**
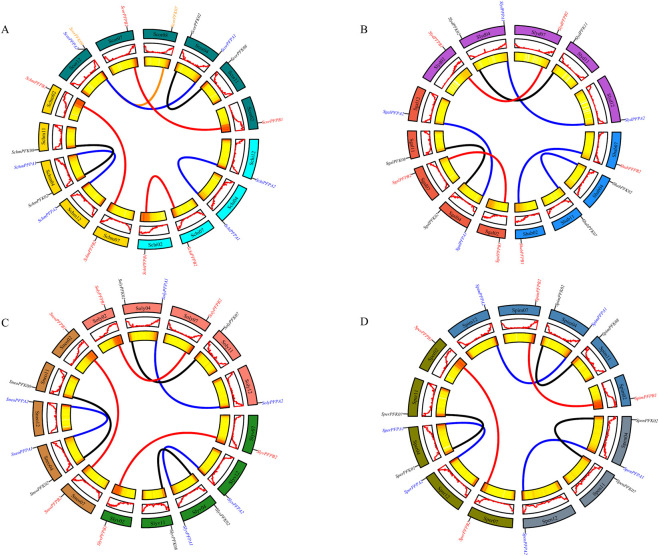
Collinearity analysis of *PFK* genes in *Solanum* species. The red lines, blue lines, orange lines, and black lines show the syntenic gene pairs of *PFK* genes. **(A)** *S.* chilense, *S. chmielewskii*, and *S. corneliomulleri*. **(B)** *S. habrochaites*, *S. galapagense*, and *S. lycopersicoides*. **(C)** *S. lycopersicum*, *S. lycopersicum var. cerasiforme*, and *S. neorickii*. **(D)** S. pennellii, S. peruvianum, and *S. pimpinellifolium*.

Synteny analysis within tomato species revealed the presence of multiple collinear gene pairs (Figure 5). In cultivated tomato (*S. lycopersicum*), three pairs of collinear genes were identified: *SolyPFPB1/SolyPFPB2*, *SolyPFK02/SolyPFK07*, and *SolyPFPA1/SolyPFPA2*. Two collinear pairs were detected in *S. habrochaites* (*ShabPFPB1/ShabPFPB2*, *ShabPFK02/ShabPFK07*) and in *S. chilense* (*SchiPFPB1/SchiPFPB2*, *SchiPFPA1/SchiPFPA2*). In *S. chmielewskii*, three pairs were identified (*SchmPFPB1/SchmPFPB2*, *SchmPFK02/SchmPFK08*, *SchmPFPA1/SchmPFPA2*), whereas *S. corneliomulleri* contained four pairs (*ScorPFPB1/ScorPFPB2*, *ScorPFK02/ScorPFK08*, *ScorPFPA1/ScorPFPA2*, *ScorPFK07/ScorPFK09*). Three collinear pairs were also identified in *S. galapagense* (*SgalPFPB1/SgalPFPB2*, *SgalPFK02/SgalPFK08*, *SgalPFPA1/SgalPFPA2*), *S. lycopersicoides* (*SlydPFPB1/SlydPFPB2*, *SlydPFK02/SlydPFK11*, *SlydPFPA1/SlydPFPA2*), *S. lycopersicum* var. *cerasiforme* (*SlyvPFPB1/SlyvPFPB2*, *SlyvPFK02/SlyvPFK08*, *SlyvPFPA1/SlyvPFPA2*), *S. neorickii* (*SneoPFPB1/SneoPFPB2*, *SneoPFK02/SneoPFK08*, *SneoPFPA1/SneoPFPA2*), *S. peruvianum* (*SperPFPB1/SperPFPB2*, *SperPFK01/SperPFK07*, *SperPFPA1/SperPFPA2*), and *S. pimpinellifolium* (*SpimPFPB1/SpimPFPB2*, *SpimPFK02/SpimPFK08*, *SpimPFPA1/SpimPFPA2*). In contrast, *S. pennellii* contained only two collinear pairs (*SpenPFK02/SpenPFK07*, *SpenPFPA1/SpenPFPA2*). Overall, most tomato species contained three collinear gene pairs, typically consisting of one PFK subfamily pair and two PFP subfamily pairs. All collinear gene pairs were located on different chromosomes, indicating that *PFK* gene expansion in tomato species mainly occurred through segmental duplication during evolution.

**FIGURE 6:**
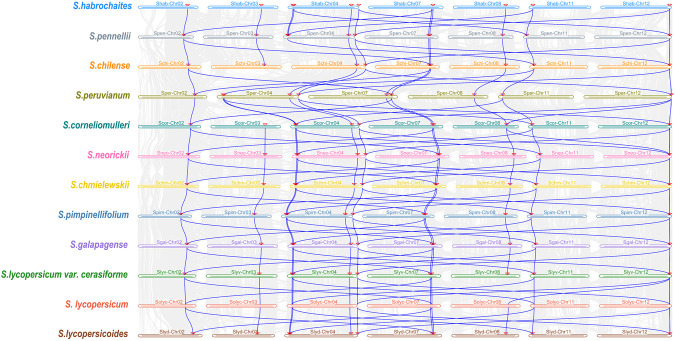
Collinearity analysis of *PFK* genes among Solanum species. The gray lines in the background show the collinear blocks within tomato and other plant genomes, while the blue lines highlight the syntenic gene pairs of *PFK* genes.

Synteny analysis among *Solanum* species (Figure 6) revealed that homologous segments of *PFK* genes were present across all species, indicating that these regions are highly conserved during evolution. Within these regions, multiple genes showed orthologous counterparts in all species, with most maintaining a consistent order of arrangement, suggesting that they originated from a common ancestor. The synteny analysis thus highlights both conservation and variation in the pan-genome structure of *Solanum*, with structural variations potentially contributing to differences in gene regulation and function. Interestingly, unlike other tomato species, *S. peruvianum* lacks *PFK* genes on Chr03, suggesting that its glycolytic function may be compensated by isoenzymes or paralogous genes located on other chromosomes to sustain essential metabolic activities. As PFK is a key rate-limiting enzyme in glycolysis, the loss of *PFK* genes is expected to markedly reduce glycolytic flux, leading to decreased sugar consumption and increased sugar accumulation.

**FIGURE 7:**
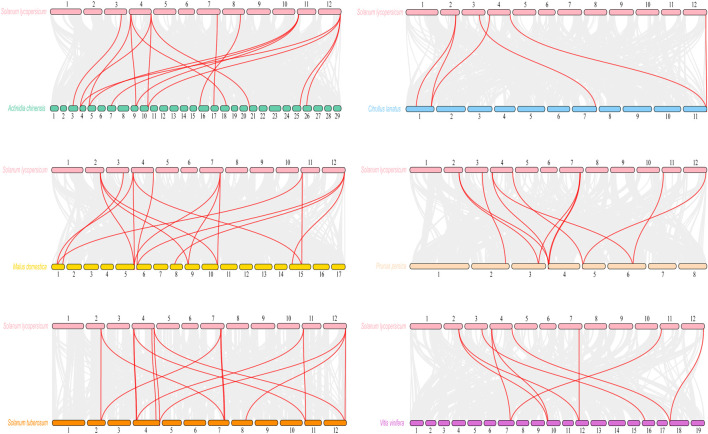
Collinearity analysis of *PFK* genes among different species. The gray lines in the background show the collinear blocks within tomato and other plant genomes, while the red lines highlight the syntenic gene pairs of *PFK* genes.

To further explore the evolutionary relationships among *PFK* gene family members, a comparative synteny map was constructed for *AchPFK*, *ClPFK*, *MdPFK*, *PpPFK*, *StPFK*, *VvPFK*, and *SlPFK* (Figure 7). The results showed that orthologous *PFK* gene pairs were present across kiwifruit, watermelon, apple, peach, potato, grape, and tomato. Specifically, *PFK* genes in apple, peach, and grape exhibited synteny with *PFK* members located on tomato Chr02, Chr03, Chr04, Chr07, Chr11, and Chr12; *PFK* genes in kiwifruit showed synteny with those on tomato Chr03, Chr04, Chr07, Chr08, Chr11, and Chr12; *PFK* genes in watermelon were syntenic with those on tomato Chr02, Chr03, Chr04, and Chr12; and *PFK* genes in potato corresponded to syntenic regions on tomato Chr02, Chr04, Chr07, Chr11, and Chr12. These results indicate that *PFK* genes associated with fruit glycolysis and sugar accumulation in tomato are mainly distributed across Chr02, Chr03, Chr04, Chr07, Chr08, Chr11, and Chr12. Furthermore, 16 orthologous gene pairs were identified between *AchPFK* and *SlPFK*, *MdPFK* and *SlPFK*, as well as *StPFK* and *SlPFK*; six orthologous pairs were found between *ClPFK* and *SlPFK*, 11 pairs between *PpPFK* and *SlPFK*, and nine pairs between *VvPFK* and *SlPFK*. This suggests that *PFK* genes in kiwifruit, apple, and potato share a closer evolutionary relationship with those in tomato.

Gene duplication is an effective mechanism for organisms to acquire new genes and maintain gene functionality. Thus, duplication plays a crucial role in gene family expansion during evolution. In this study, the substitution rates of homologous *PFK* gene pairs were calculated to investigate their evolutionary origins (Table 3). The results showed that in cultivated tomato, three homologous gene pairs (*SolyPFK02*/*SolyPFK07*, *SolyPFPA1*/*SolyPFPA2*, and *SolyPFPB1*/*SolyPFPB2*) were all derived from segmental duplication events. Similarly, *S. chmielewskii*, *S. galapagense*, *S. lycopersicoides*, *S. lycopersicum var. cerasiforme*, *S. neorickii*, *S. peruvianum*, and *S. pimpinellifolium* each contained three homologous gene pairs, all located on Chr02, Chr04, Chr07, Chr11, and Chr12. *S. habrochaites*, *S. chilense*, and *S. pennellii* contained two homologous gene pairs, whereas *S. corneliomulleri* harbored four homologous pairs distributed across Chr02, Chr04, Chr07, Chr08, Chr11, and Chr12. All of these homologous pairs were found to originate from segmental duplication events.

**TABLE 3:** Ka/Ks analysis of the *PFK* gene.

Species	Chr	Duplicate gene pairs	Ka	Ks	Ka/Ks	Duplicated type
*S. lycopersicum*	SolyChr02/SolyChr07	*SolyPFPB1*/*SolyPFPB2*	0.13	1.62	0.08	Segmental
	SolyChr04/SolyChr11	*SolyPFK02*/*SolyPFK07*	0.09	0.51	0.18	Segmental
	SolyChr04/SolyChr12	*SolyPFPA1*/*SolyPFPA2*	0.05	0.56	0.08	Segmental
*S. habrochaites*	ShabChr02/ShabChr07	*ShabPFPB1*/*ShabPFPB2*	0.16	1.60	0.10	Segmental
	ShabChr04/ShabChr11	*ShabPFK02*/*ShabPFK07*	0.09	0.52	0.17	Segmental
*S. chilense*	SchiChr02/SchiChr07	*SchiPFPB1*/*SchiPFPB2*	0.12	1.42	0.09	Segmental
	SchiChr04/SchiChr12	*SchiPFPA1*/*SchiPFPA2*	0.05	0.54	0.09	Segmental
*S. chmielewskii*	SchmChr02/SchmChr07	*SchmPFPB1*/*SchmPFPB2*	0.13	1.61	0.08	Segmental
	SchmChr04/SchmChr11	*SchmPFK02*/*SchmPFK08*	0.09	0.52	0.18	Segmental
	SchmChr04/SchmChr12	*SchmPFPA1*/*SchmPFPA2*	0.05	0.54	0.09	Segmental
*S. corneliomulleri*	ScorChr02/ScorChr07	*ScorPFPB1*/*ScorPFPB2*	0.13	1.63	0.08	Segmental
	ScorChr04/ScorChr11	*ScorPFK02*/*ScorPFK08*	0.09	0.52	0.18	Segmental
	ScorChr04/ScorChr12	*ScorPFPA1*/*ScorPFPA2*	0.05	0.54	0.09	Segmental
	ScorChr08/ScorChr12	*ScorPFK07*/*ScorPFK09*	0.08	0.63	0.13	Segmental
*S. galapagense*	SgalChr02/SgalChr07	*SgalPFPB1*/*SgalPFPB2*	0.11	2.32	0.05	Segmental
	SgalChr04/SgalChr11	*SgalPFK02*/*SgalPFK08*	0.09	0.66	0.13	Segmental
	SgalChr04/SgalChr12	*SgalPFPA1*/*SgalPFPA2*	0.04	0.60	0.08	Segmental
*S. lycopersicoides*	SlydChr02/SlydChr07	*SlydPFPB1*/*SlydPFPB2*	0.14	1.51	0.09	Segmental
	SlydChr04/SlydChr11	*SlydPFK02*/*SlydPFK11*	0.09	0.47	0.19	Segmental
	SlydChr04/SlydChr12	*SlydPFPA1*/*SlydPFPA2*	0.06	0.56	0.11	Segmental
*S. lycopersicum var. cerasiforme*	SlyvChr02/SlyvChr07	*SlyvPFPB1*/*SlyvPFPB2*	0.13	1.62	0.08	Segmental
	SlyvChr04/SlyvChr11	*SlyvPFK02*/*SlyvPFK08*	0.09	0.51	0.18	Segmental
	SlyvChr04/SlyvChr12	*SlyvPFPA1*/*SlyvPFPA2*	0.05	0.56	0.08	Segmental
*S. neorickii*	SneoChr02/SneoChr07	*SneoPFPB1*/*SneoPFPB2*	0.13	1.61	0.08	Segmental
	SneoChr04/SneoChr11	*SneoPFK02*/*SneoPFK08*	0.09	0.52	0.17	Segmental
	SneoChr04/SneoChr12	*SneoPFPA1*/*SneoPFPA2*	0.05	0.54	0.09	Segmental
*S. pennellii*	SpenChr04/SpenChr11	*SpenPFK02*/*SpenPFK07*	0.09	0.53	0.16	Segmental
	SpenChr04/SpenChr12	*SpenPFPA1*/*SpenPFPA2*	0.05	0.52	0.09	Segmental
*S. peruvianum*	SperChr02/SperChr07	*SperPFPB1*/*SperPFPB2*	0.13	1.44	0.09	Segmental
	SperChr04/SperChr11	*SperPFK01*/*SperPFK07*	0.09	0.54	0.17	Segmental
	SperChr04/SperChr12	*SperPFPA1*/*SperPFPA2*	0.05	0.54	0.09	Segmental
*S. pimpinellifolium*	SpimChr02/SpimChr07	*SpimPFPB1*/*SpimPFPB2*	0.13	1.60	0.08	Segmental
	SpimChr04/SpimChr11	*SpimPFK02*/*SpimPFK08*	0.09	0.54	0.17	Segmental
	SpimChr04/SpimChr12	*SpimPFPA1*/*SpimPFPA2*	0.05	0.56	0.08	Segmental

The ratio of nonsynonymous (Ka) to synonymous (Ks) substitution rates (Ka/Ks) is a widely used parameter in molecular evolution for evaluating whether genes or protein-coding sequences are under selective pressure. A Ka/Ks ratio close to 1 indicates neutral evolution, a ratio greater than 1 suggests positive selection, and a ratio less than 1 reflects purifying selection (Hurst, 2002). In the present study, all *PFK* genes exhibited Ka/Ks values less than 1, indicating that members of the *PFK* gene family have experienced strong purifying selection throughout evolution.

### 3.6 Cis-acting element analysis of PFK gene promoters

**FIGURE 8:**
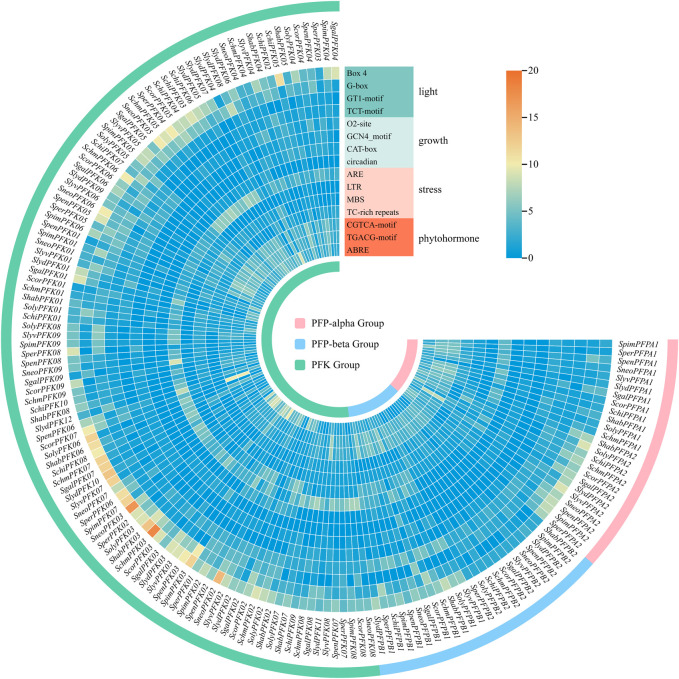
Cis-acting element distribution of *PFK* genes. The number of cis-acting elements is indicated by a color scale ranging from dark blue to dark red. Blue signifies the lowest number, while red signifies the highest number.

Cis-acting element analysis of tomato *PFK* gene family members revealed four major categories based on their functions: light-responsive elements, plant growth and development-related elements, stress-responsive elements, and phytohormone-responsive elements (Figure 8). Among these, light-responsive elements (Box 4, G-box, GT1-motif, TCT-motif) were the most abundant, with a total of 2036 elements identified. Among these, Box 4 elements were the most numerous (954), followed by G-box elements (527). *SchmPFK03* contained the highest number of Box 4 elements, followed by *SneoPFK03*, while *SpimPFK03* harbored the most G-box elements, followed by *ShabPFK03*. Box 4 represents a DNA module involved in light responses, and G-box is a cis-acting element associated with light responsiveness. As Box 4 elements were detected in all *PFK* genes, it can be inferred that these genes may participate in light response pathways, thereby regulating photosynthesis and promoting tomato growth and development. In addition, 277 GT1-motif and 278 TCT-motif elements were identified. A total of 395 growth- and development-related elements were detected across all *PFK* genes, including 165 O2-site elements (involved in zein metabolism regulation), 87 CAT-box elements (associated with meristem expression), 82 GCN4_motif elements (associated with endosperm expression), and 61 circadian elements (involved in circadian growth regulation). Regarding stress responsiveness, 1020 cis-elements were identified, consisting of four main types: ARE, LTR, MBS, and TC-rich repeats. ARE elements, which regulate binding of transcription factors and anaerobic induction, were the most abundant (526). MBS elements (drought-responsive) ranked second (196), followed by low-temperature (LTR) elements (152) and TC-rich repeats involved in defense and stress responses (146). Phytohormone-responsive elements were also highly represented, with 1,342 elements in total. The main types included abscisic acid-responsive elements (ABRE, 432) and methyl jasmonate-responsive elements (CGTCA-motif and TGACG-motif, 455 each). Abscisic acid regulates tomato growth and enhances stress tolerance, while methyl jasmonate promotes defense responses, conferring disease resistance. Collectively, these findings suggest that *PFK* genes play critical roles in regulating plant growth and development, as well as enhancing stress resistance, thereby contributing to tomato growth and adaptation under adverse environmental conditions.

### 3.7 Expression analysis of PFK genes

**FIGURE 9:**
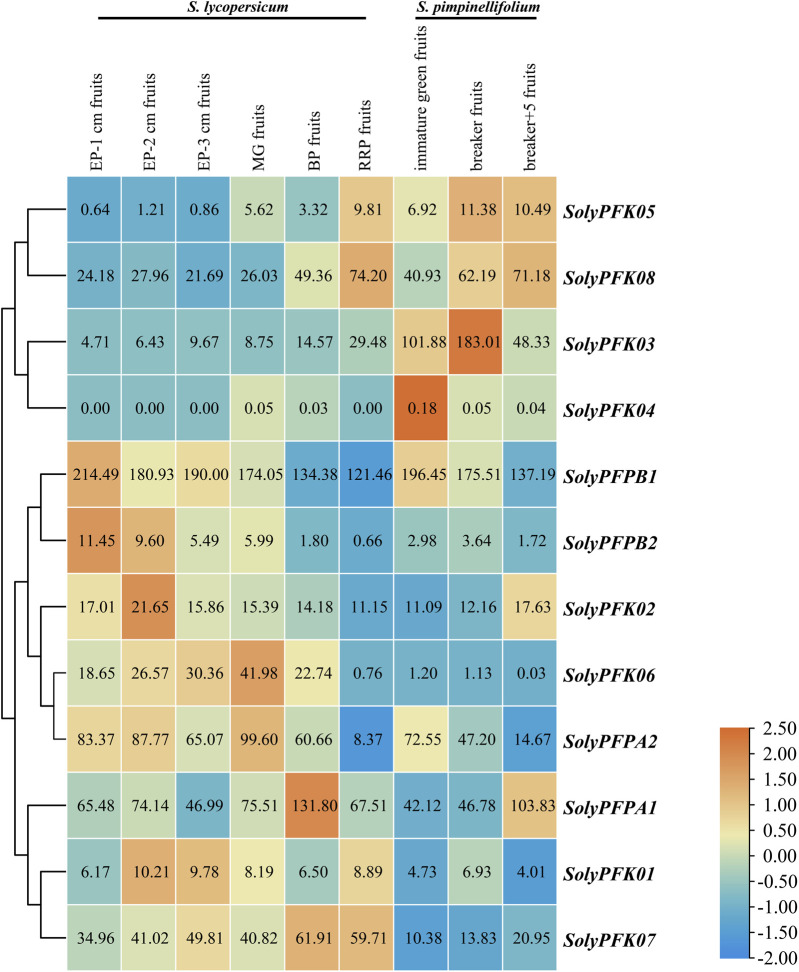
Heatmap of *PFK* genes expression. Dark blue, light blue, yellow, light red and dark red are used to represent gene expression levels. Blue indicates weak gene expression, red indicates strong gene expression. EP:Expansion stage; MG:Mature green stage; BP:Breaker stage; RRP:Red ripening stage.

To investigate the potential biological functions of *PFK* genes during tomato fruit development, their expression profiles were analyzed using RNA-seq data from different developmental stages of Heinz and currant tomatoes. As shown in Figure 9, *SolyPFK01*, *SolyPFK02*, *SolyPFK04*, *SolyPFK05*, and *SolyPFPB2* exhibited consistently low expression levels throughout all growth stages in Heinz tomato. Similarly, in currant tomato, *SolyPFK01*, *SolyPFK02*, *SolyPFK04*, *SolyPFK05*, *SolyPFK06*, and *SolyPFPB2* maintained low expression levels across all stages. Notably, the expression of *SolyPFK04* was negligible in both tomato varieties.

*SolyPFPB1* exhibited the highest expression levels across all developmental stages, suggesting its extensive involvement in regulating tomato fruit development. Concurrently, the expression of *SolyPFPB1* displayed a decreasing trend as fruit development progressed. This pattern may be attributed to the predominance of glycolysis during early fruit development, where high *PFK* expression and enhanced enzymatic activity facilitate the catabolism of photosynthates to provide energy for cell growth. Conversely, as the fruit transitions from the breaker to the red ripe stage, the downregulation of *PFK* expression and reduced enzymatic activity lead to a slower glycolytic rate. This reduction in sugar consumption favors sugar accumulation, resulting in a rapid increase in total sugar content. Furthermore, certain genes exhibited differential expression trends between the two tomato varieties, likely reflecting genetic variations in fruit development and metabolic regulation strategies. In Heinz tomato, *SolyPFPA1* exhibited high expression levels during fruit development, peaking at the breaker stage; however, its expression significantly decreased at the red ripe stage, dropping to approximately 50% of the level observed at the breaker stage. Similarly, *SolyPFPA2* maintained relatively high expression from the expansion to the mature green stage in Heinz tomato, but subsequently declined at the breaker stage and dropped further at the red ripe stage to less than 10% of the breaker-stage level. This downregulation may be attributed to reduced glycolytic activity and enhanced sugar accumulation during fruit maturation. Conversely, in currant tomato, *SolyPFPA1* displayed a continuous upward trend, reaching its peak at the red ripe stage with expression levels more than double those of the preceding stage. In contrast, *SolyPFPA2* showed a gradual downward trend, reaching its lowest point at the red ripe stage, where expression was approximately one-third of that in the previous stage. Although both belong to the PFP-α subfamily, *SolyPFPA1* and *SolyPFPA2* exhibited opposite expression patterns in currant tomato, reflecting potential functional differentiation in sugar metabolism pathways during ripening. It is speculated that *SolyPFPA1* may be associated with higher metabolic activity or specific ripening regulatory mechanisms, whereas the continuous downregulation of *SolyPFPA2* likely reflects an adaptive regulation for sugar preservation and accumulation. Notably, *SolyPFK08* maintained high expression levels with a continuous upward trend across all developmental stages in both tomato varieties, suggesting a stable and conserved regulatory role for this gene. Collectively, these results demonstrate that *PFK* genes exhibit stage-specific and cultivar-specific expression characteristics, highlighting their crucial regulatory functions in tomato fruit growth, glycolysis, and sugar accumulation.

### 3.8 Protein–protein interaction analysis of PFK proteins

**FIGURE 10:**
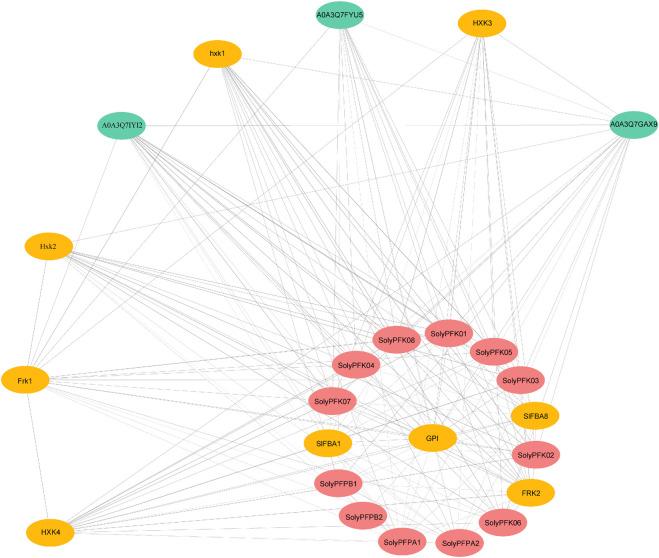
Interaction network of PFK proteins.

To further elucidate the potential biological functions of PFK proteins, we predicted protein interactions among 12 PFK members using the STRING database (Figure 10). Several proteins exhibited direct interactions, such as SolyPFPA1 with SolyPFPB1, SolyPFPA1 with SolyPFPA2, and SolyPFPA2 with SolyPFPB1. In addition, SolyPFKs were found to interact with other transcriptional regulatory proteins, including GPI, hxk1, Hxk2, HXK3, HXK4, Frk1, FRK2, and SlFBA1. The HXK proteins belong to the hexokinase family, which are key enzymes in sugar metabolism with both catalytic and glucose-sensing functions. Specifically, hxk1 and Hxk2 repress the expression of photosynthesis-related genes while promoting the expression of metabolism-related genes, thereby coordinating carbon source utilization. Hxk2 has also been implicated in regulating crop growth, development, and stress responses, while HXK3 and HXK4 are primarily participate in fundamental metabolic processes. FRK proteins, members of the fructokinase family, specifically catalyze the phosphorylation of fructose to generate fructose-6-phosphate (F6P), with Frk1 and FRK2 being responsible for this activity. SlFBA1 and GPI are also key enzymes in the glycolytic pathway, suggesting that PFK proteins may cooperate with these enzymes to regulate sugar metabolism in tomato fruit. Interestingly, several unannotated proteins, such as A0A3Q7FYU5, A0A3Q7IYI2, and A0A3Q7GAX9, were also identified in the interaction network. Although their functions remain unclear, they exhibited direct or indirect interactions with PFK proteins, implying potential roles in metabolic regulation. Collectively, these findings indicate that PFK proteins have diverse functions and may act in concert with other proteins to regulate tomato growth, development, and sugar metabolism.

### 3.9 qRT-PCR analysis of PFK genes

**FIGURE 11:**
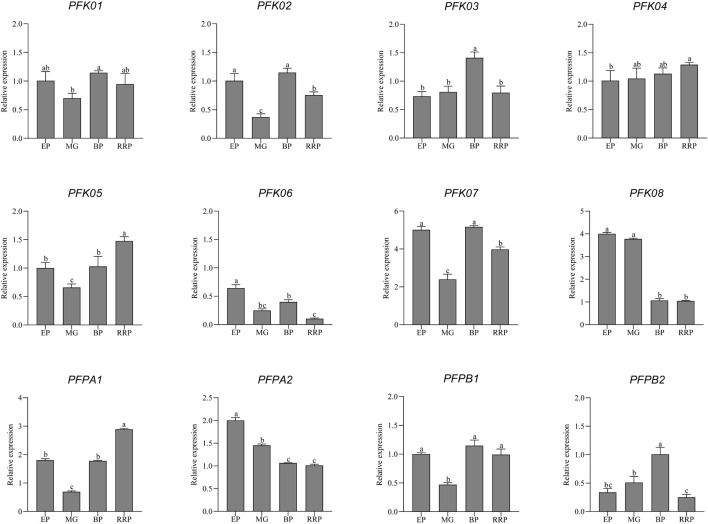
qRT-PCR analysis of *PFK* genes expression in tomato fruits. EP:Expansion stage; MG:Mature green stage; BP:Breaker stage; RRP:Red ripening stage. The standard deviation determined from three biological replicates is shown in error bars. The statistical significance of differences in PFK gene expression levels was determined using one-way ANOVA (p < 0.05). Significant differences (p < 0.05) are indicated by different lowercase letters.

Phosphofructokinase (PFK) plays a crucial regulatory role during fruit growth and development. In this study, the expression patterns of *PFK* genes were analyzed in cultivated tomato fruits at different developmental stages using qRT-PCR (Figure 11). Most genesincluding *PFK01*, *PFK02*, *PFK06*, *PFK07*, *PFK08*, *PFPA2*, *PFPB1*, and *PFPB2*, exhibited high expression levels during the fruit expansion stage, which gradually decreased by the red ripe stage. This trend suggests that these genes function in glycolysis during the early stages of fruit development to promote growth, whereas their expression declines in later stages, reducing glycolytic activity and thereby facilitating sugar accumulation. Notably, *PFK02*, *PFK06*, *PFK07*, *PFK08*, *PFPA2*, and *PFPB2* showed significant expression differences between the expansion and red ripe stages, highlighting their prominent regulatory roles during these periods. In contrast, *PFK03, PFK04, PFK05,* and *PFPA1* displayed low expression in early fruit growth but higher expression at maturity, suggesting their involvement in glycolysis throughout fruit development, contributing to the biosynthesis of metabolic compounds. Among them, *PFK04* expression gradually increased across developmental stages, implying enhanced glycolytic activity, while *PFK03* exhibited its highest expression specifically at the breaker stage, significantly higher than at other stages. Moreover, *PFK04, PFK05,* and *PFPA1* were markedly more highly expressed in the red ripe stage compared to the expansion stage, whereas *PFK03* showed no significant difference between these two stages. Interestingly, *PFPA2* expression continuously declined throughout fruit development, indicating its primary role in glycolysis during early fruit growth, supplying energy for cell proliferation and expansion. As the fruit matured, decreased *PFPA2* activity slowed glycolysis, reducing sugar consumption and promoting sugar accumulation.

An integrated analysis of RNA-seq and qRT-PCR data reveals that different PFK family members exhibit distinct phase-specific patterns and functional differentiation during tomato fruit development. Specifically, *PFK05* was significantly upregulated during the middle-to-late stages of fruit development across multiple tomato varieties, suggesting its potential involvement in metabolic regulation associated with fruit ripening. *PFK07* displayed a distinct stage-specific expression pattern characterized by a decline from the expansion to the mature green stage, a significant increase at the breaker stage, and a subsequent downregulation at the red ripe stage, indicating it may play a key regulatory role during the breaker stage. Conversely, *PFPA2* expression exhibited a progressive declining trend throughout the entire fruit development period. This pattern, consistent with its RNA-seq profile in currant tomato, implies that *PFPA2* may contribute to reducing the glycolytic rate during maturation, thereby facilitating sugar accumulation. In summary, *PFK* genes play crucial regulatory roles in the glycolytic pathway and sugar accumulation through differential expression during tomato fruit development.

## 4 Discussion

Wild relatives of tomato are widely distributed, highly adaptable, and exhibit extensive genetic variation, thereby providing valuable genetic diversity and resources for tomato breeding. The assembly and release of the tomato super-pangenome have substantially deepened our understanding of tomato genome evolution, elucidating the evolutionary relationships between wild and cultivated species and facilitating the identification of beneficial allelic variations from wild germplasm. Glycolysis, as a fundamental metabolic pathway, provides energy for cellular metabolism, with phosphofructokinase (PFK) functioning as a key rate-limiting enzyme and major regulatory node in plants. Despite its central role, studies on the *PFK* gene family in tomato remain scarce. In this study, we performed a comprehensive identification of *PFK* genes across the *Solanum* species, encompassing 12 tomato species. A total of 156 *PFK* genes were identified, including 108 genes in the PFK subfamily and 48 genes in the PFP subfamily. These genes were distributed across seven chromosomes. Specifically, PFK subfamily members were located on chromosomes 3, 4, 7, 8, 11, and 12; the PFP-α subfamily members were found on chromosomes 4 and 12; and the PFP-β subfamily members were distributed on chromosomes 2 and 7. This suggests that gene duplication is a key driving force behind the expansion of this gene family. The members within each subfamily originated from a common ancestral chromosome, and these genes were retained after duplication. Therefore, the chromosomal distribution of the *PFK* gene family members reveals a potential mechanism for achieving functional diversification through duplication and divergence, providing a genetic basis for the regulation of sugar metabolism in tomato across different tissues and environmental conditions.

In this study, we analyzed the phylogenetic relationships of *PFK* genes from six representative species, including tomato, kiwifruit, potato, apple, maize, and grape. The *PFK* gene family was divided into two major subgroups, PFK and PFP. Genes within the same subfamily exhibited high sequence homology, suggesting that duplicated homologs likely originated from a common ancestor and may share similar biological functions (Lynch and Conery, 2000). *MePFK03* and *OsPFK05* both belong to the PFK_A subfamily, and their expression levels are upregulated under waterlogging stress, indicating that they play important regulatory roles in metabolism under hypoxic conditions in cassava and rice (Wang et al., 2021). Gene duplication is widely recognized as a major driver of rapid gene family expansion and evolutionary innovation. Through chromosomal localization and gene structure analyses, we revealed duplication events that contributed to the expansion of the *Solanum* pangenome during evolution. A total of 34 pairs of segmental duplications were identified among the 156 *PFK* genes, whereas no tandem duplications were detected, indicating that segmental duplication has played a dominant role in the expansion of the *PFK* gene family. Evolutionary pressure analysis further showed that the Ka/Ks ratios of all 34 duplicated gene pairs were less than 1, suggesting that *PFK* genes in tomato have been subjected to strong purifying selection, thereby maintaining the structural and functional stability of this family throughout long-term evolution. This strong purifying selection indicates that the PFK gene family plays an important role in maintaining the efficiency and robustness of basic metabolism and the glycolytic pathway in tomato. Collinearity analysis within the *Solanum* species demonstrated that homologous *PFK* regions are conserved across all tomato species, with multiple orthologous genes displaying highly syntenic arrangements, suggesting their derivation from a common ancestor. Comparative collinearity among tomato and other representative species revealed that kiwifruit, apple, and potato shared the largest number of syntenic *PFK* homologs with tomato, implying closer evolutionary relationships among these species. In addition, these crops may share common mechanisms in the regulation of sugar metabolism, which provides new genetic resources and theoretical support for systematically identifying *PFK* homologous genes and achieving coordinated genetic improvement across multiple crops through comparative genomics approaches.

Cis-regulatory elements act as crucial molecular switches in transcriptional regulation, particularly under biotic and abiotic stresses, and play pivotal roles in controlling gene expression (Lescot et al., 2002). To investigate the potential biological functions of tomato *PFK* genes, we analyzed cis-elements within the 2,000 bp upstream promoter regions. The results revealed that *PFK* genes harbor a wide range of elements associated with plant growth and development, hormone responsiveness, and stress adaptation. These include elements involved in meristem-specific expression, hormone regulation, and responses to abiotic stresses such as low temperature and drought. Notably, the majority of *PFK* family members were predicted to respond to multiple phytohormones, particularly abscisic acid (ABA) and methyl jasmonate (MeJA), both of which are key regulators in plant stress signaling pathways. ABA and MeJA are known to enhance plant tolerance by increasing antioxidant enzyme activity, scavenging reactive oxygen species, and promoting the accumulation of osmoprotectants, thereby mitigating stress-induced damage. Accordingly, it is plausible that *PFK* genes participate in abiotic stress responses through ABA- and MeJA-mediated signaling pathways, contributing to improved stress tolerance in tomato. This indicates that *PFK* genes in tomato not only participate in fundamental growth and developmental processes throughout the plant’s life cycle but also play crucial roles in responding to various environmental challenges.

Phosphofructokinases (PFKs) have been implicated in diverse biological processes, including tissue development, sugar accumulation in fruits, and responses to abiotic stresses. For example, *PFP1-1*, *PFP1-2*, and *PFP1-3* regulate starch deposition and soluble sugar synthesis in rice grains (Chen et al., 2020a; Duan et al., 2016). In maize, *ZmPFK2* and *ZmPFK6* are highly expressed in roots, stems, and leaves, and actively respond to abiotic stresses such as drought and low temperature (Fu et al., 2025). Similarly, *MePFK03* expression is significantly induced in cassava roots under waterlogging stress (Wang et al., 2021). The potential biological functions of *PFK* genes during tomato fruit development were investigated in this study. Based on RNA-seq and qRT-PCR data, the expression patterns of *PFK* genes were analyzed at different stages of tomato fruit development. The results indicate that *PFK* genes play important regulatory roles in fruit growth, glycolysis, and sugar accumulation in tomato.

Fruit development is a complex process in which plants convert photosynthetic products into edible tissues. In this study, we observed that *PFK* genes exhibit differential expression patterns across various stages of tomato fruit development, which is highly correlated with their pivotal roles in regulating fruit sugar content. We hypothesize that *PFK* genes may modulate the metabolic flux of the glycolytic pathway, thereby affecting the transport efficiency of photoassimilates from source to sink, their metabolic conversion within the fruit, and ultimately, the level of sugar accumulation.

## 5 Conclusion

Phosphofructokinase (PFK), as a major rate-limiting enzyme and regulatory node in glycolysis, plays essential roles in plant growth, stress responses, fruit development, and sugar accumulation. In this study, a total of 156 *PFK* genes were systematically identified across 12 *Solanum* species, which were unevenly distributed on seven chromosomes. Phylogenetic analysis divided these genes into two major subfamilies, PFK and PFP (further classified into PFP-α and PFP-β). Within each subfamily, motif compositions were highly conserved, whereas certain motifs were unique to specific subfamilies, suggesting functional diversification of PFK proteins. Collinearity analysis indicated that segmental duplication was the main driver of *PFK* family expansion, and Ka/Ks analysis revealed that strong purifying selection has shaped their evolutionary trajectory. Cis-acting element analysis identified numerous motifs associated with development, abiotic stress, and hormone responses, implying that *PFK* genes contribute to tomato growth and stress adaptation. Protein–protein interaction analysis further suggested that PFK proteins cooperate with other metabolic regulators to promote fruit growth and development. Moreover, RNA-seq and qRT-PCR data demonstrated stage-specific expression of *PFK* genes during tomato fruit development, highlighting their involvement in glycolysis and sugar accumulation. Collectively, this study provides a comprehensive characterization of the *PFK* gene family in *Solanum*, offering valuable insights into their evolutionary patterns, regulatory mechanisms, and potential biological functions in abiotic stress responses and fruit development.

## Data Availability

The RNA-seq data for this study are publicly available in the Tomato Functional Genome Database (http://132.236.156.160/cgi-bin/TFGD/digital/home.cgi). This data can be obtained by searching the gene IDs listed in Table 2. The corresponding dataset accession numbers are S0586, S0587, S0588, S0589, S0590, S05891, S05894, S05895, and S05896.
