# Supplementary material for: Identification of the PFK gene family in Solanum species and expression analysis in the fruitof Solanum lycopersicum
Source: Front Genet. 2026 Mar 9;17:1738448. doi: 10.3389/fgene.2026.1738448 (PMC13006092; doi:10.3389/fgene.2026.1738448)
Supplement: Supplementary file 1 [file Table1.doc]

**Supplementary Tables**

**Table S1.** Physicochemical properties of amino acid sequences encoded by members of the PFK gene family in *S. habrochaites*.

| Gene ID | Gene name | Chromosome location | Amino acid | Molecular  weight | Isoelectrc  point | Gravy | Subcellular  localization | Protein secondary structure | | |
| --- | --- | --- | --- | --- | --- | --- | --- | --- | --- | --- |
|  |  |  |  |  |  |  |  | a | b | c |
| Shab02g017660.1 | *ShabPFPB1* | Shab-Chr02 | 569 | 61797.86 | 6.01 | -0.124 | chlo | 45.52% | 6.85% | 33.22% |
| Shab03g014140.1 | *ShabPFK01* | Shab-Chr03 | 539 | 59571.97 | 8.71 | -0.263 | chlo | 30.61% | 7.05% | 41.19% |
| Shab04g005380.1 | *ShabPFK02* | Shab-Chr04 | 536 | 58652.34 | 8.73 | -0.121 | cyto | 31.53% | 7.46% | 44.22% |
| Shab04g006410.1 | *ShabPFK03* | Shab-Chr04 | 456 | 50461.35 | 6.21 | -0.163 | chlo | 33.99% | 8.11% | 38.60% |
| Shab04g019320.1 | *ShabPFK04* | Shab-Chr04 | 427 | 47523.69 | 5.28 | -0.262 | cysk | 33.26% | 6.79% | 39.81% |
| Shab04g019340.1 | *ShabPFK05* | Shab-Chr04 | 327 | 36651.63 | 5.22 | -0.165 | cysk | 37.92% | 7.03% | 37.00% |
| Shab04g027150.1 | *ShabPFPA1* | Shab-Chr04 | 579 | 63657.05 | 6.90 | -0.155 | cyto | 44.91% | 5.01% | 35.06% |
| Shab07g013370.1 | *ShabPFPB2* | Shab-Chr07 | 461 | 50752.40 | 8.24 | -0.243 | cyto | 43.38% | 6.94% | 34.92% |
| Shab08g010860.1 | *ShabPFK06* | Shab-Chr08 | 465 | 51034.18 | 6.54 | -0.152 | cysk | 33.76% | 8.17% | 38.92% |
| Shab11g004200.1 | *ShabPFK07* | Shab-Chr11 | 532 | 58363.82 | 6.76 | -0.103 | cyto | 31.95% | 7.71% | 42.86% |
| Shab12g018670.1 | *ShabPFPA2* | Shab-Chr12 | 610 | 66503.32 | 7.24 | -0.151 | cyto | 46.23% | 5.41% | 33.61% |
| Shab12g018790.1 | *ShabPFK08* | Shab-Chr12 | 485 | 53766.01 | 5.99 | -0.315 | cyto | 33.40% | 7.22% | 41.24% |

**Table S2.** Physicochemical properties of amino acid sequences encoded by members of the PFK gene family in *S. chilense*.

| Gene ID | Gene name | Chromosome location | Amino acid | Molecular  weight | Isoelectrc  point | Gravy | Subcellular  localization | Protein secondary structure | | |
| --- | --- | --- | --- | --- | --- | --- | --- | --- | --- | --- |
|  |  |  |  |  |  |  |  | a | b | c |
| Schi02g018300.1 | *SchiPFPB1* | Schi-Chr02 | 569 | 61821.92 | 6.01 | -0.125 | cyto | 45.52% | 5.80% | 32.86% |
| Schi03g013980.1 | *SchiPFK01* | Schi-Chr03 | 539 | 59620.03 | 8.71 | -0.272 | chlo | 32.84% | 7.24% | 40.82% |
| Schi04g017730.1 | *SchiPFK02* | Schi-Chr04 | 499 | 55047.19 | 5.44 | -0.266 | cysk | 31.66% | 7.01% | 42.89% |
| Schi04g017760.1 | *SchiPFK03* | Schi-Chr04 | 288 | 31882.18 | 5.99 | -0.173 | cysk | 30.90% | 6.94% | 44.79% |
| Schi04g017780.1 | *SchiPFK04* | Schi-Chr04 | 356 | 39407.86 | 6.37 | -0.281 | cysk | 32.87% | 9.27% | 38.20% |
| Schi04g017810.1 | *SchiPFK05* | Schi-Chr04 | 478 | 52886.18 | 5.99 | -0.227 | cysk | 33.68% | 6.69% | 40.38% |
| Schi04g017850.1 | *SchiPFK06* | Schi-Chr04 | 380 | 41557.12 | 6.00 | -0.159 | cysk | 37.37% | 6.84% | 37.37% |
| Schi04g026200.1 | *SchiPFPA1* | Schi-Chr04 | 643 | 70517.95 | 7.08 | -0.086 | cyto | 47.90% | 5.44% | 32.97% |
| Schi07g010790.1 | *SchiPFK07* | Schi-Chr07 | 510 | 56269.04 | 6.25 | -0.244 | cysk | 36.47% | 6.08% | 40.78% |
| Schi07g011930.1 | *SchiPFPB2* | Schi-Chr07 | 554 | 60121.00 | 7.55 | -0.171 | chlo | 44.04% | 6.86% | 32.13% |
| Schi08g010210.1 | *SchiPFK08* | Schi-Chr08 | 491 | 54036.43 | 7.22 | -0.251 | cysk | 31.36% | 6.31% | 44.20% |
| Schi11g004950.1 | *SchiPFK09* | Schi-Chr11 | 529 | 57991.37 | 6.47 | -0.113 | cyto | 30.81% | 7.37% | 44.61% |
| Schi12g019110.1 | *SchiPFPA2* | Schi-Chr12 | 617 | 67198.08 | 6.98 | -0.147 | cyto | 46.52% | 6.00% | 33.39% |
| Schi12g019230.1 | *SchiPFK10* | Schi-Chr12 | 485 | 53768.02 | 6.18 | -0.329 | cyto | 32.16% | 7.01% | 42.06% |

**Table S3.** Physicochemical properties of amino acid sequences encoded by members of the PFK gene family in *S. chmielewskii*.

| Gene ID | Gene name | Chromosome location | Amino acid | Molecular  weight | Isoelectrc  point | Gravy | Subcellular  localization | Protein secondary structure | | |
| --- | --- | --- | --- | --- | --- | --- | --- | --- | --- | --- |
|  |  |  |  |  |  |  |  | a | b | c |
| Schm02g017750.1 | *SchmPFPB1* | Schm-Chr02 | 569 | 61807.89 | 6.01 | -0.126 | cyto | 44.82% | 5.80% | 33.92% |
| Schm03g013930.2 | *SchmPFK01* | Schm-Chr03 | 539 | 59624.00 | 8.71 | -0.264 | chlo | 32.28% | 7.42% | 42.86% |
| Schm04g005110.1 | *SchmPFK02* | Schm-Chr04 | 538 | 58842.50 | 8.64 | -0.113 | cyto | 30.11% | 8.74% | 42.57% |
| Schm04g005790.1 | *SchmPFK03* | Schm-Chr04 | 470 | 52230.61 | 6.57 | -0.127 | chlo | 34.89% | 8.51% | 37.66% |
| Schm04g017310.1 | *SchmPFK04* | Schm-Chr04 | 499 | 55005.19 | 5.32 | -0.252 | cyto | 30.66% | 7.41% | 42.08% |
| Schm04g017340.1 | *SchmPFK05* | Schm-Chr04 | 291 | 32646.08 | 5.99 | -0.167 | cysk | 30.93% | 9.97% | 40.89% |
| Schm04g024980.1 | *SchmPFPA1* | Schm-Chr04 | 616 | 67287.11 | 6.64 | -0.112 | cyto | 45.62% | 5.19% | 35.23% |
| Schm07g011290.2 | *SchmPFK06* | Schm-Chr07 | 509 | 56159.97 | 6.37 | -0.230 | cysk | 36.35% | 6.68% | 40.67% |
| Schm07g012410.1 | *SchmPFPB2* | Schm-Chr07 | 554 | 60105.94 | 7.56 | -0.170 | chlo | 44.04% | 6.14% | 33.94% |
| Schm08g009810.1 | *SchmPFK07* | Schm-Chr08 | 491 | 54050.46 | 7.64 | -0.253 | cysk | 30.14% | 6.52% | 45.21% |
| Schm11g003710.1 | *SchmPFK08* | Schm-Chr11 | 532 | 58358.76 | 6.47 | -0.100 | cyto | 30.08% | 7.33% | 45.11% |
| Schm12g017400.1 | *SchmPFPA2* | Schm-Chr12 | 617 | 67277.21 | 7.23 | -0.146 | cyto | 45.87% | 6.32% | 33.06% |
| Schm12g017530.1 | *SchmPFK09* | Schm-Chr12 | 485 | 53768.02 | 6.18 | -0.329 | cyto | 32.16% | 7.01% | 42.06% |

**Table S4.** Physicochemical properties of amino acid sequences encoded by members of the PFK gene family in *S. corneliomulleri*.

| Gene ID | Gene name | Chromosome location | Amino acid | Molecular  weight | Isoelectrc  point | Gravy | Subcellular  localization | Protein secondary structure | | |
| --- | --- | --- | --- | --- | --- | --- | --- | --- | --- | --- |
|  |  |  |  |  |  |  |  | a | b | c |
| Scor02g017130.1 | *ScorPFPB1* | Scor-Chr02 | 569 | 61807.89 | 6.01 | -0.126 | cyto | 44.99% | 5.80% | 33.57% |
| Scor03g014460.3 | *ScorPFK01* | Scor-Chr03 | 539 | 59654.08 | 8.81 | -0.267 | chlo | 33.02% | 6.68% | 42.30% |
| Scor04g005170.1 | *ScorPFK02* | Scor-Chr04 | 538 | 58828.47 | 8.64 | -0.114 | cyto | 31.23% | 8.36% | 42.75% |
| Scor04g005830.1 | *ScorPFK03* | Scor-Chr04 | 414 | 46138.73 | 6.47 | -0.138 | chlo | 30.68% | 8.21% | 40.10% |
| Scor04g017240.1 | *ScorPFK04* | Scor-Chr04 | 499 | 54975.16 | 5.26 | -0.248 | cysk | 32.46% | 7.01% | 42.08% |
| Scor04g017260.1 | *ScorPFK05* | Scor-Chr04 | 387 | 42476.14 | 5.95 | -0.201 | cysk | 35.66% | 5.17% | 40.31% |
| Scor04g024820.1 | *ScorPFPA1* | Scor-Chr04 | 616 | 67301.18 | 6.76 | -0.112 | cyto | 46.59% | 5.52% | 33.93% |
| Scor07g012040.1 | *ScorPFK06* | Scor-Chr07 | 501 | 55486.49 | 8.39 | -0.192 | cysk | 34.93% | 6.59% | 40.32% |
| Scor07g013060.1 | *ScorPFPB2* | Scor-Chr07 | 554 | 60104.96 | 7.92 | -0.170 | chlo | 45.13% | 6.50% | 31.95% |
| Scor08g009580.1 | *ScorPFK07* | Scor-Chr08 | 500 | 55025.73 | 6.71 | -0.196 | cyto | 33.40% | 7.40% | 40.40% |
| Scor11g004520.1 | *ScorPFK08* | Scor-Chr11 | 532 | 58315.73 | 6.44 | -0.098 | cyto | 30.26% | 8.08% | 43.05% |
| Scor12g018930.1 | *ScorPFPA2* | Scor-Chr12 | 617 | 67198.08 | 6.98 | -0.147 | cyto | 46.52% | 6.00% | 33.39% |
| Scor12g019050.1 | *ScorPFK09* | Scor-Chr12 | 485 | 53768.02 | 6.18 | -0.329 | cyto | 32.16% | 7.01% | 42.06% |

**Table S5.** Physicochemical properties of amino acid sequences encoded by members of the PFK gene family in *S. galapagense*.

| Gene ID | Gene name | Chromosome location | Amino acid | Molecular  weight | Isoelectrc  point | Gravy | Subcellular  localization | Protein secondary structure | | |
| --- | --- | --- | --- | --- | --- | --- | --- | --- | --- | --- |
|  |  |  |  |  |  |  |  | a | b | c |
| Sgal02g019250.1 | *SgalPFPB1* | Sgal-Chr02 | 569 | 61807.89 | 6.01 | -0.126 | cyto | 44.64% | 6.15% | 33.57% |
| Sgal03g015500.1 | *SgalPFK01* | Sgal-Chr03 | 539 | 59714.13 | 8.71 | -0.265 | chlo | 31.91% | 6.68% | 42.49% |
| Sgal04g005180.1 | *SgalPFK02* | Sgal-Chr04 | 538 | 58858.45 | 8.55 | -0.129 | cyto | 30.30% | 8.55% | 42.38% |
| Sgal04g005990.1 | *SgalPFK03* | Sgal-Chr04 | 456 | 50513.51 | 6.31 | -0.161 | chlo | 31.80% | 8.11% | 40.13% |
| Sgal04g017570.1 | *SgalPFK04* | Sgal-Chr04 | 495 | 54387.57 | 5.26 | -0.226 | cyto | 31.31% | 7.27% | 41.21% |
| Sgal04g017600.1 | *SgalPFK05* | Sgal-Chr04 | 390 | 42974.68 | 5.81 | -0.177 | cysk | 37.95% | 5.64% | 41.28% |
| Sgal04g025310.1 | *SgalPFPA1* | Sgal-Chr04 | 616 | 67301.18 | 6.76 | -0.112 | cyto | 46.59% | 5.52% | 33.93% |
| Sgal07g012370.2 | *SgalPFK06* | Sgal-Chr07 | 509 | 56188.95 | 6.25 | -0.230 | cysk | 33.99% | 7.47% | 42.04% |
| Sgal07g013480.1 | *SgalPFPB2* | Sgal-Chr07 | 554 | 60090.89 | 7.56 | -0.169 | chlo | 45.85% | 5.23% | 33.75% |
| Sgal08g011150.1 | *SgalPFK07* | Sgal-Chr08 | 500 | 55053.79 | 6.92 | -0.197 | cyto | 33.80% | 7.80% | 41.60% |
| Sgal11g004540.1 | *SgalPFK08* | Sgal-Chr11 | 532 | 58304.75 | 6.47 | -0.097 | cyto | 29.51% | 7.71% | 44.74% |
| Sgal12g020130.1 | *SgalPFPA2* | Sgal-Chr12 | 617 | 67268.13 | 6.79 | -0.152 | cyto | 45.22% | 5.35% | 35.49% |
| Sgal12g020250.1 | *SgalPFK09* | Sgal-Chr12 | 485 | 53741.99 | 6.22 | -0.333 | cyto | 31.34% | 6.39% | 42.89% |

**Table S6.** Physicochemical properties of amino acid sequences encoded by members of the PFK gene family in *S. lycopersicoides*.

| Gene ID | Gene name | Chromosome location | Amino acid | Molecular  weight | Isoelectrc  point | Gravy | Subcellular  localization | Protein secondary structure | | |
| --- | --- | --- | --- | --- | --- | --- | --- | --- | --- | --- |
|  |  |  |  |  |  |  |  | a | b | c |
| Slyd02g016960.1 | *SlydPFPB1* | Slyd-Chr02 | 570 | 61950.09 | 5.91 | -0.107 | cyto | 44.56% | 6.49% | 31.93% |
| Slyd03g009840.1 | *SlydPFK01* | Slyd-Chr03 | 518 | 57527.46 | 8.08 | -0.272 | chlo | 33.40% | 7.53% | 41.89% |
| Slyd04g005310.1 | *SlydPFK02* | Slyd-Chr04 | 526 | 57511.06 | 8.95 | -0.101 | cysk | 30.99% | 7.98% | 43.73% |
| Slyd04g006020.1 | *SlydPFK03* | Slyd-Chr04 | 456 | 50515.48 | 6.28 | -0.148 | chlo | 33.77% | 7.24% | 40.13% |
| Slyd04g016490.1 | *SlydPFK04* | Slyd-Chr04 | 495 | 54666.91 | 5.27 | -0.267 | cyto | 30.30% | 7.07% | 43.84% |
| Slyd04g016510.1 | *SlydPFK05* | Slyd-Chr04 | 300 | 32617.73 | 5.05 | -0.266 | cyto | 39.67% | 7.33% | 38.00% |
| Slyd04g016520.1 | *SlydPFK06* | Slyd-Chr04 | 497 | 54601.01 | 5.48 | -0.182 | cyto | 35.81% | 7.24% | 39.24% |
| Slyd04g016530.1 | *SlydPFK07* | Slyd-Chr04 | 429 | 47433.76 | 5.24 | -0.205 | cysk | 31.47% | 5.59% | 42.19% |
| Slyd04g016540.1 | *SlydPFK08* | Slyd-Chr04 | 425 | 46643.75 | 5.59 | -0.207 | cysk | 33.41% | 7.76% | 40.47% |
| Slyd04g024530.1 | *SlydPFPA1* | Slyd-Chr04 | 644 | 70739.20 | 6.90 | -0.111 | cyto | 46.12% | 6.21% | 33.54% |
| Slyd07g010840.1 | *SlydPFK09* | Slyd-Chr07 | 509 | 56024.83 | 6.33 | -0.212 | cysk | 33.79% | 6.48% | 41.85% |
| Slyd07g011890.1 | *SlydPFPB2* | Slyd-Chr07 | 539 | 58867.42 | 6.68 | -0.184 | chlo | 42.30% | 6.31% | 37.11% |
| Slyd08g009550.1 | *SlydPFK10* | Slyd-Chr08 | 500 | 55168.84 | 6.71 | -0.222 | cyto | 32.00% | 8.40% | 41.00% |
| Slyd11g006060.1 | *SlydPFK11* | Slyd-Chr11 | 531 | 58188.57 | 6.60 | -0.087 | cyto | 28.25% | 7.53% | 43.69% |
| Slyd12g017230.1 | *SlydPFPA2* | Slyd-Chr12 | 617 | 67325.27 | 7.23 | -0.153 | cyto | 46.19% | 4.21% | 35.66% |
| Slyd12g017350.1 | *SlydPFK12* | Slyd-Chr12 | 481 | 53338.53 | 6.03 | -0.309 | cyto | 34.30% | 7.90% | 40.12% |

**Table S7.** Physicochemical properties of amino acid sequences encoded by members of the PFK gene family in *S. lycopersicum var. cerasiforme*.

| Gene ID | Gene name | Chromosome location | Amino acid | Molecular  weight | Isoelectrc  point | Gravy | Subcellular  localization | Protein secondary structure | | |
| --- | --- | --- | --- | --- | --- | --- | --- | --- | --- | --- |
|  |  |  |  |  |  |  |  | a | b | c |
| Slyv02g018880.1 | *SlyvPFPB1* | Slyv-Chr02 | 569 | 61765.81 | 6.01 | -0.134 | cyto | 44.64% | 6.15% | 33.57% |
| Slyv03g014720.1 | *SlyvPFK01* | Slyv-Chr03 | 539 | 59714.13 | 8.71 | -0.265 | chlo | 31.91% | 6.68% | 42.49% |
| Slyv04g005180.1 | *SlyvPFK02* | Slyv-Chr04 | 533 | 58461.02 | 8.43 | -0.133 | cyto | 30.21% | 8.07% | 43.15% |
| Slyv04g006010.1 | *SlyvPFK03* | Slyv-Chr04 | 456 | 50513.51 | 6.31 | -0.161 | chlo | 31.80% | 8.11% | 40.13% |
| Slyv04g017280.1 | *SlyvPFK04* | Slyv-Chr04 | 499 | 54836.99 | 5.20 | -0.233 | cyto | 31.66% | 7.21% | 42.69% |
| Slyv04g017310.1 | *SlyvPFK05* | Slyv-Chr04 | 390 | 43000.72 | 5.81 | -0.191 | cysk | 36.15% | 6.15% | 42.31% |
| Slyv04g025150.1 | *SlyvPFPA1* | Slyv-Chr04 | 616 | 67301.18 | 6.76 | -0.112 | cyto | 46.59% | 5.52% | 33.93% |
| Slyv07g012350.2 | *SlyvPFK06* | Slyv-Chr07 | 509 | 56188.95 | 6.25 | -0.230 | cysk | 33.99% | 7.47% | 42.04% |
| Slyv07g013400.1 | *SlyvPFPB2* | Slyv-Chr07 | 554 | 60090.89 | 7.56 | -0.169 | chlo | 45.49% | 6.32% | 33.03% |
| Slyv08g011090.1 | *SlyvPFK07* | Slyv-Chr08 | 500 | 55053.79 | 6.92 | -0.197 | cyto | 33.80% | 7.80% | 41.60% |
| Slyv11g004390.1 | *SlyvPFK08* | Slyv-Chr11 | 532 | 58365.79 | 6.47 | -0.104 | cyto | 30.45% | 7.33% | 43.80% |
| Slyv12g019700.1 | *SlyvPFPA2* | Slyv-Chr12 | 617 | 67267.19 | 7.23 | -0.153 | cyto | 46.35% | 5.51% | 34.85% |
| Slyv12g019820.1 | *SlyvPFK09* | Slyv-Chr12 | 485 | 53800.03 | 6.12 | -0.339 | cyto | 31.96% | 8.04% | 40.21% |

**Table S8.** Physicochemical properties of amino acid sequences encoded by members of the PFK gene family in *S. neorickii*.

| Gene ID | Gene name | Chromosome location | Amino acid | Molecular  weight | Isoelectrc  point | Gravy | Subcellular  localization | Protein secondary structure | | |
| --- | --- | --- | --- | --- | --- | --- | --- | --- | --- | --- |
|  |  |  |  |  |  |  |  | a | b | c |
| Sneo02g018750.1 | *SneoPFPB1* | Sneo-Chr02 | 569 | 61807.89 | 6.01 | -0.126 | cyto | 44.82% | 5.98% | 33.57% |
| Sneo03g013870.1 | *SneoPFK01* | Sneo-Chr03 | 539 | 59650.08 | 8.81 | -0.268 | chlo | 30.06% | 6.86% | 45.27% |
| Sneo04g004720.1 | *SneoPFK02* | Sneo-Chr04 | 538 | 58842.50 | 8.64 | -0.114 | cyto | 30.67% | 8.18% | 43.87% |
| Sneo04g005730.1 | *SneoPFK03* | Sneo-Chr04 | 456 | 50556.62 | 6.43 | -0.147 | chlo | 31.80% | 8.99% | 39.47% |
| Sneo04g018140.1 | *SneoPFK04* | Sneo-Chr04 | 499 | 55018.27 | 5.45 | -0.251 | cyto | 32.26% | 7.01% | 42.89% |
| Sneo04g018170.1 | *SneoPFK05* | Sneo-Chr04 | 390 | 43211.24 | 6.00 | -0.152 | cysk | 34.87% | 4.87% | 41.28% |
| Sneo04g025780.1 | *SneoPFPA1* | Sneo-Chr04 | 616 | 67301.18 | 6.76 | -0.112 | cyto | 46.59% | 5.52% | 33.93% |
| Sneo07g011460.1 | *SneoPFK06* | Sneo-Chr07 | 509 | 56183.93 | 6.37 | -0.244 | cysk | 33.99% | 6.88% | 42.04% |
| Sneo07g012510.1 | *SneoPFPB2* | Sneo-Chr07 | 554 | 60105.94 | 7.56 | -0.170 | chlo | 44.04% | 6.14% | 33.94% |
| Sneo08g010300.1 | *SneoPFK07* | Sneo-Chr08 | 515 | 56813.80 | 8.39 | -0.213 | cysk | 33.59% | 6.99% | 41.75% |
| Sneo11g004420.1 | *SneoPFK08* | Sneo-Chr11 | 532 | 58391.81 | 6.47 | -0.101 | cyto | 29.14% | 6.77% | 46.43% |
| Sneo12g018180.1 | *SneoPFPA2* | Sneo-Chr12 | 617 | 67249.16 | 7.23 | -0.149 | cyto | 46.19% | 5.02% | 35.01% |
| Sneo12g018310.1 | *SneoPFK09* | Sneo-Chr12 | 485 | 53780.08 | 6.18 | -0.318 | cysk | 30.93% | 6.39% | 43.92% |

**Table S9.** Physicochemical properties of amino acid sequences encoded by members of the PFK gene family in *S. pennellii*.

| Gene ID | Gene name | Chromosome location | Amino acid | Molecular  weight | Isoelectrc  point | Gravy | Subcellular  localization | Protein secondary structure | | |
| --- | --- | --- | --- | --- | --- | --- | --- | --- | --- | --- |
|  |  |  |  |  |  |  |  | a | b | c |
| Sopen02g025800.1 | *SpenPFPB1* | Spen-Chr02 | 569 | 61827.88 | 6.01 | -0.129 | chlo | 44.99% | 6.15% | 32.51% |
| Sopen03g024340.2 | *SpenPFK01* | Spen-Chr03 | 539 | 59582.01 | 8.71 | -0.265 | chlo | 30.61% | 6.49% | 42.86% |
| Sopen04g006390.1 | *SpenPFK02* | Spen-Chr04 | 538 | 58794.45 | 8.64 | -0.112 | cyto | 32.34% | 9.11% | 40.89% |
| Sopen04g007240.1 | *SpenPFK03* | Spen-Chr04 | 456 | 50493.45 | 6.31 | -0.158 | chlo | 32.46% | 7.89% | 40.35% |
| Sopen04g028780.1 | *SpenPFK04* | Spen-Chr04 | 486 | 53562.81 | 5.60 | -0.181 | cyto | 34.77% | 7.41% | 39.30% |
| Sopen04g036520.1 | *SpenPFPA1* | Spen-Chr04 | 616 | 67315.25 | 6.90 | -0.113 | cyto | 46.43% | 5.52% | 34.09% |
| Sopen07g023710.1 | *SpenPFK05* | Spen-Chr07 | 490 | 54106.70 | 6.65 | -0.244 | cyto | 34.49% | 7.14% | 40.41% |
| Sopen07g024610.1 | *SpenPFPB2* | Spen-Chr07 | 554 | 60121.94 | 7.56 | -0.174 | chlo | 44.58% | 6.50% | 33.57% |
| Sopen08g019800.1 | *SpenPFK06* | Spen-Chr08 | 273 | 30139.41 | 7.09 | -0.183 | cysk | 28.57% | 10.26% | 37.73% |
| Sopen11g005230.1 | *SpenPFK07* | Spen-Chr11 | 532 | 58339.75 | 6.36 | -0.098 | cyto | 30.08% | 7.52% | 44.17% |
| Sopen12g031660.1 | *SpenPFPA2* | Spen-Chr12 | 617 | 67281.22 | 7.23 | -0.151 | cyto | 45.87% | 5.83% | 33.39% |
| Sopen12g031780.1 | *SpenPFK08* | Spen-Chr12 | 485 | 53777.12 | 6.18 | -0.305 | cyto | 33.20% | 8.25% | 40.00% |

**Table S10.** Physicochemical properties of amino acid sequences encoded by members of the PFK gene family in *S. peruvianum*.

| Gene ID | Gene name | Chromosome location | Amino acid | Molecular  weight | Isoelectrc  point | Gravy | Subcellular  localization | Protein secondary structure | | |
| --- | --- | --- | --- | --- | --- | --- | --- | --- | --- | --- |
|  |  |  |  |  |  |  |  | a | b | c |
| Sper02g017010.1 | *SperPFPB1* | Sper-Chr02 | 569 | 61821.92 | 6.01 | -0.125 | cyto | 43.94% | 5.80% | 33.39% |
| Sper04g005380.1 | *SperPFK01* | Sper-Chr04 | 538 | 58784.41 | 8.64 | -0.111 | cyto | 31.04% | 8.36% | 44.05% |
| Sper04g006220.1 | *SperPFK02* | Sper-Chr04 | 456 | 50530.54 | 6.43 | -0.161 | chlo | 33.11% | 7.02% | 41.01% |
| Sper04g017840.1 | *SperPFK03* | Sper-Chr04 | 499 | 55018.23 | 5.31 | -0.233 | cyto | 32.87% | 6.41% | 42.48% |
| Sper04g017860.1 | *SperPFK04* | Sper-Chr04 | 390 | 42833.56 | 5.74 | -0.196 | cysk | 36.67% | 5.13% | 41.28% |
| Sper04g025440.1 | *SperPFPA1* | Sper-Chr04 | 643 | 70503.88 | 6.90 | -0.086 | cyto | 46.97% | 6.07% | 32.35% |
| Sper07g011870.1 | *SperPFK05* | Sper-Chr07 | 509 | 56113.88 | 6.25 | -0.233 | cysk | 32.81% | 7.07% | 43.03% |
| Sper07g015980.1 | *SperPFPB2* | Sper-Chr07 | 554 | 60075.92 | 7.56 | -0.165 | chlo | 44.04% | 5.78% | 35.20% |
| Sper08g010280.1 | *SperPFK06* | Sper-Chr08 | 500 | 55067.86 | 7.21 | -0.199 | cyto | 31.80% | 8.20% | 41.00% |
| Sper11g004320.1 | *SperPFK07* | Sper-Chr11 | 532 | 58294.72 | 6.47 | -0.092 | cyto | 29.89% | 8.46% | 43.42% |
| Sper12g021710.1 | *SperPFK08* | Sper-Chr12 | 485 | 53768.02 | 6.18 | -0.329 | cyto | 32.16% | 7.01% | 42.06% |
| Sper12g021830.1 | *SperPFPA2* | Sper-Chr12 | 617 | 67212.15 | 7.23 | -0.147 | cyto | 47.00% | 5.19% | 33.23% |

**Table S11.** Physicochemical properties of amino acid sequences encoded by members of the PFK gene family in *S. pimpinellifolium*.

| Gene ID | Gene name | Chromosome location | Amino acid | Molecular  weight | Isoelectrc  point | Gravy | Subcellular  localization | Protein secondary structure | | |
| --- | --- | --- | --- | --- | --- | --- | --- | --- | --- | --- |
|  |  |  |  |  |  |  |  | a | b | c |
| Spim02g017070.1 | *SpimPFPB1* | Spim-Chr02 | 569 | 61807.89 | 6.01 | -0.126 | cyto | 44.99% | 5.80% | 33.57% |
| Spim03g015340.2 | *SpimPFK01* | Spim-Chr03 | 539 | 59714.13 | 8.71 | -0.265 | chlo | 31.91% | 6.68% | 42.49% |
| Spim04g005350.1 | *SpimPFK02* | Spim-Chr04 | 538 | 58858.45 | 8.55 | -0.129 | cyto | 30.30% | 8.55% | 42.38% |
| Spim04g006000.1 | *SpimPFK03* | Spim-Chr04 | 456 | 50503.47 | 6.31 | -0.159 | chlo | 31.80% | 8.33% | 40.13% |
| Spim04g017690.1 | *SpimPFK04* | Spim-Chr04 | 495 | 54399.58 | 5.38 | -0.223 | cyto | 33.33% | 6.46% | 41.41% |
| Spim04g017720.1 | *SpimPFK05* | Spim-Chr04 | 391 | 43084.80 | 5.81 | -0.199 | cysk | 35.29% | 5.88% | 41.69% |
| Spim04g025440.1 | *SpimPFPA1* | Spim-Chr04 | 616 | 67301.18 | 6.76 | -0.112 | cyto | 46.59% | 5.52% | 33.93% |
| Spim07g012180.1 | *SpimPFK06* | Spim-Chr07 | 509 | 56188.95 | 6.25 | -0.230 | cysk | 33.99% | 7.47% | 42.04% |
| Spim07g013270.1 | *SpimPFPB2* | Spim-Chr07 | 554 | 60090.89 | 7.56 | -0.169 | chlo | 44.58% | 5.96% | 33.57% |
| Spim08g010730.1 | *SpimPFK07* | Spim-Chr08 | 491 | 54036.43 | 7.22 | -0.249 | cysk | 30.55% | 8.55% | 42.97% |
| Spim11g004400.1 | *SpimPFK08* | Spim-Chr11 | 532 | 58338.77 | 6.47 | -0.099 | cyto | 32.33% | 7.52% | 42.48% |
| Spim12g020130.1 | *SpimPFPA2* | Spim-Chr12 | 617 | 67267.19 | 7.23 | -0.153 | cyto | 46.35% | 5.51% | 34.85% |
| Spim12g020260.1 | *SpimPFK09* | Spim-Chr12 | 485 | 53741.99 | 6.22 | -0.333 | cyto | 31.34% | 6.39% | 42.89% |
